# Associations of genetics, behaviors, and life course circumstances with a novel aging and healthspan measure: Evidence from the Health and Retirement Study

**DOI:** 10.1371/journal.pmed.1002827

**Published:** 2019-06-18

**Authors:** Zuyun Liu, Xi Chen, Thomas M. Gill, Chao Ma, Eileen M. Crimmins, Morgan E. Levine

**Affiliations:** 1 Department of Pathology, Yale School of Medicine, New Haven, Connecticut, United States of America; 2 Department of Health Policy and Management, Yale School of Public Health, New Haven, Connecticut, United States of America; 3 Department of Economics, Yale University, New Haven, Connecticut, United States of America; 4 Department of Internal Medicine, Yale School of Medicine, New Haven, Connecticut, United States of America; 5 School of Public Health, Southeast University, Nanjing, Jiangsu Province, China; 6 Davis School of Gerontology, University of Southern California, Los Angeles, California, United States of America; 7 Department of Epidemiology, Yale School of Public Health, New Haven, Connecticut, United States of America; University og Southern Denmark, DENMARK

## Abstract

**Background:**

An individual’s rate of aging directly influences his/her susceptibility to morbidity and mortality. Thus, quantifying aging and disentangling how various factors coalesce to produce between-person differences in the rate of aging, have important implications for potential interventions. We recently developed and validated a novel multi-system-based aging measure, Phenotypic Age (PhenoAge), which has been shown to capture mortality and morbidity risk in the full US population and diverse subpopulations. The aim of this study was to evaluate associations between PhenoAge and a comprehensive set of factors, including genetic scores, childhood and adulthood circumstances, and health behaviors, to determine the relative contributions of these factors to variance in this aging measure.

**Methods and findings:**

Based on data from 2,339 adults (aged 51+ years, mean age 69.4 years, 56% female, and 93.9% non-Hispanic white) from the US Health and Retirement Study, we calculated PhenoAge and evaluated the multivariable associations for a comprehensive set of factors using 2 innovative approaches—Shapley value decomposition (the Shapley approach hereafter) and hierarchical clustering. The Shapley approach revealed that together all 11 study domains (4 childhood and adulthood circumstances domains, 5 polygenic score [PGS] domains, and 1 behavior domain, and 1 demographic domain) accounted for 29.2% (bootstrap standard error = 0.003) of variance in PhenoAge after adjustment for chronological age. Behaviors exhibited the greatest contribution to PhenoAge (9.2%), closely followed by adulthood adversity, which was suggested to contribute 9.0% of the variance in PhenoAge. Collectively, the PGSs contributed 3.8% of the variance in PhenoAge (after accounting for chronological age). Next, using hierarchical clustering, we identified 6 distinct subpopulations based on the 4 childhood and adulthood circumstances domains. Two of these subpopulations stood out as disadvantaged, exhibiting significantly higher PhenoAges on average. Finally, we observed a significant gene-by-environment interaction between a previously validated PGS for coronary artery disease and the seemingly most disadvantaged subpopulation, suggesting a multiplicative effect of adverse life course circumstances coupled with genetic risk on phenotypic aging. The main limitations of this study were the retrospective nature of self-reported circumstances, leading to possible recall biases, and the unrepresentative racial/ethnic makeup of the population.

**Conclusions:**

In a sample of US older adults, genetic, behavioral, and socioenvironmental circumstances during childhood and adulthood account for about 30% of differences in phenotypic aging. Our results also suggest that the detrimental effects of disadvantaged life course circumstances for health and aging may be further exacerbated among persons with genetic predisposition to coronary artery disease. Finally, our finding that behaviors had the largest contribution to PhenoAge highlights a potential policy target. Nevertheless, further validation of these findings and identification of causal links are greatly needed.

## Abstract 2

Morgan Levine and colleagues reveal that behavioral and socioeconomic factors contribute more to aging than genetic factors in a new model

## Author summary

**Why was this study done?:**

- Identifying factors that account for between-person differences in the rate of aging has important implications for potential interventions.
- We recently developed and validated a novel multi-system-based aging measure, Phenotypic Age (PhenoAge), which has been shown to capture mortality and morbidity risk.
- The aim of this study was to evaluate associations of a comprehensive set of factors, including genetics, behaviors, and childhood and adulthood circumstances, with PhenoAge, to determine their relative contributions to variance in this aging measure.

**What did the researchers do and find?:**

- We classified 11 domains (4 childhood and adulthood circumstances domains, 5 polygenic score domains, 1 behavior domain, and 1 demographic domain) and estimated that together they accounted for about 30% of variance in phenotypic aging.
- Behaviors exhibited the greatest contribution to variance in PhenoAge (9.2%), closely followed by adulthood adversity (9%).
- We identified subpopulations of participants, represented by shared childhood and adulthood circumstances, that exhibited either accelerated or decelerated phenotypic aging.
- Genetic predisposition to coronary artery disease further exacerbated phenotypic aging among those in the most disadvantaged subpopulation.

**What do these findings mean?:**

- Behavioral factors and socioenvironmental circumstances have the largest influence over levels of phenotypic aging. This is important given the modifiability of these factors, highlighting a potential policy target.
- Genetic predisposition may multiply the impact of socioenvironmental circumstances in a super-additive synergistic manner, suggesting risk stratification that may inform targeted intervention strategies.

## Introduction

One major driver in the pathogenesis of many chronic diseases is presumed to be aging [1,2], a complex multifactorial process characterized by increasing dysregulation and loss of function across multiple levels and systems [3]. Thus, quantifying aging and disentangling how various factors coalesce to produce between-person differences in the rate of aging have important implications for potential interventions. We recently developed and validated a novel multi-system-based aging measure, Phenotypic Age (PhenoAge), which has been shown to capture mortality and morbidity risk in the full US population and diverse subpopulations, even among healthy individuals [4,5]. PhenoAge is meant to capture age-related dysregulation and can facilitate identification of individuals at risk for a number of chronic diseases or causes of death. It can also be applied to basic and observational research, shedding light on genetic and environmental factors that alter the pace of aging.

Genetic differences are hypothesized to contribute to differential vulnerability for aging and disease, such that life span is estimated to be 20%–30% heritable [6], while twin studies suggest that fatal coronary heart disease is about 40%–50% heritable [7–9]. Nevertheless, a large proportion of the variance in age-related outcomes is presumed to be driven by environment. For instance, previous work has provided strong evidence that traumas and adversities in childhood and adulthood influence the risk of various outcomes (e.g., disease and mortality) in later life [10–13], presumably via an acceleration of the aging process [14–17]. The cumulative “wear and tear” in response to chronic stressors or deprivation experienced over the life course is thought to contribute to declines in physiological adaptation (i.e., allostatic load) that manifest as vulnerability to disease and death [18,19]. “Wear and tear” is believed to account for the observation that low socioeconomic status (SES) is often accompanied by increased risk for long-term aging-related outcomes worldwide [14,16,20–30].

To capture a more complete picture of how various factors coalesce and in turn manifest as health disparities, it is necessary to assemble a comprehensive set of life course circumstances that can be examined concurrently. While various factors, including socioenvironmental circumstances and genetics, influence health and aging, their relative contributions to aging and disease risk are uncertain. The multitude of factors defining an individual’s specific life course circumstances poses challenges for modeling the effects of individual variables and cumulative effects. Variance in health (i.e., inequality) can be influenced by circumstances throughout the life course; yet, there are variations in the level of control a person has over the circumstances shown to contribute to health and aging inequalities. One key contribution of the current research is to innovatively distinguish circumstances outside versus within the realm of one’s control. Shapley value decomposition (the Shapley approach hereafter) facilitates estimation of the share of health inequality due to circumstances with varying degrees of modifiability. By appropriately assigning contributions from sources of health inequality, the Shapley approach estimates the overall and relative importance of these sources of inequality (see details of statistical analyses in S1 Appendix). In doing so, this approach has the potential to inform the priorities of public policies aimed at alleviating health inequality.

Using data from a large sample of US older adults, including a comprehensive set of factors assessing childhood and adulthood circumstances, behaviors, and genetics, the present study calculated PhenoAge and innovatively applied 2 approaches—the Shapley approach and hierarchical clustering—to evaluate the associations between these various factors and PhenoAge. The results will inform the development of preventive interventions to promote healthy aging and reduce morbidity and mortality risk.

## Methods

### Data

**Fig 1:**
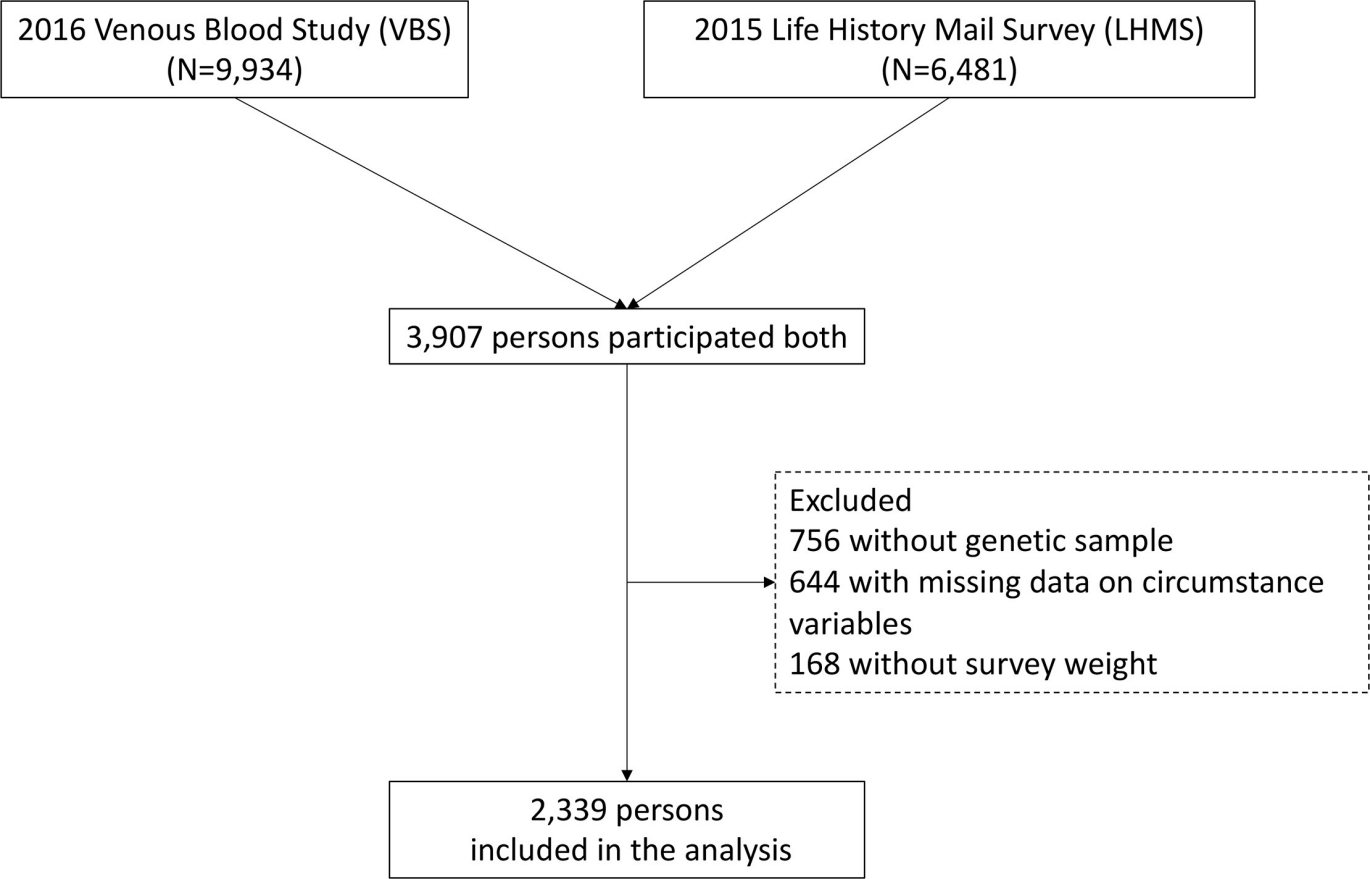
Flow chart of study participants. Genetic samples (saliva) were collected in the Enhanced Face-To-Face interview from 2006 to 2012.

The Health and Retirement Study (HRS) is an ongoing, nationally representative, biennial survey of older Americans (aged 51+ years) and their spouses, beginning in 1992 [31]. HRS is funded by the National Institute on Aging and carried out by the University of Michigan. In this study, we assembled a large array of variables from 4 components within HRS, including the core survey (1996–2016), the newly released 2015 Life History Mail Survey (LHMS), the Enhanced Face-To-Face (EFTF) interview (2006–2016), and the 2016 Venous Blood Study (VBS). A description of the 4 data sources can be found in S1 Appendix and elsewhere [31]. After restricting the sample to persons who participated in each of these sub-studies, our final analytic sample included 2,339 persons (Fig 1). Compared with persons who participated in both the 2015 LHMS and 2016 VBS but were excluded in this analysis, our analytic sample showed a similar sex ratio but was older (mean age 69.4 versus 68.3 years), more highly educated (mean number of years of school 13.9 versus 13.2 years), and more likely to be non-Hispanic white (93.9% versus 84.0%). Note that our analytic sample excluded participants who did not self-identify as either non-Hispanic white or non-Hispanic black, given that polygenic scores (PGSs) were only available for participants of European and/or African ancestry. As a result, the analytic sample in this study is not nationally representative. HRS was approved by the Institutional Review Board of the University of Michigan, and all participants provided informed consent. Data used in this study are de-identified and publicly available (http://hrsonline.isr.umich.edu).

### Childhood and adulthood circumstances

**Fig 2:**
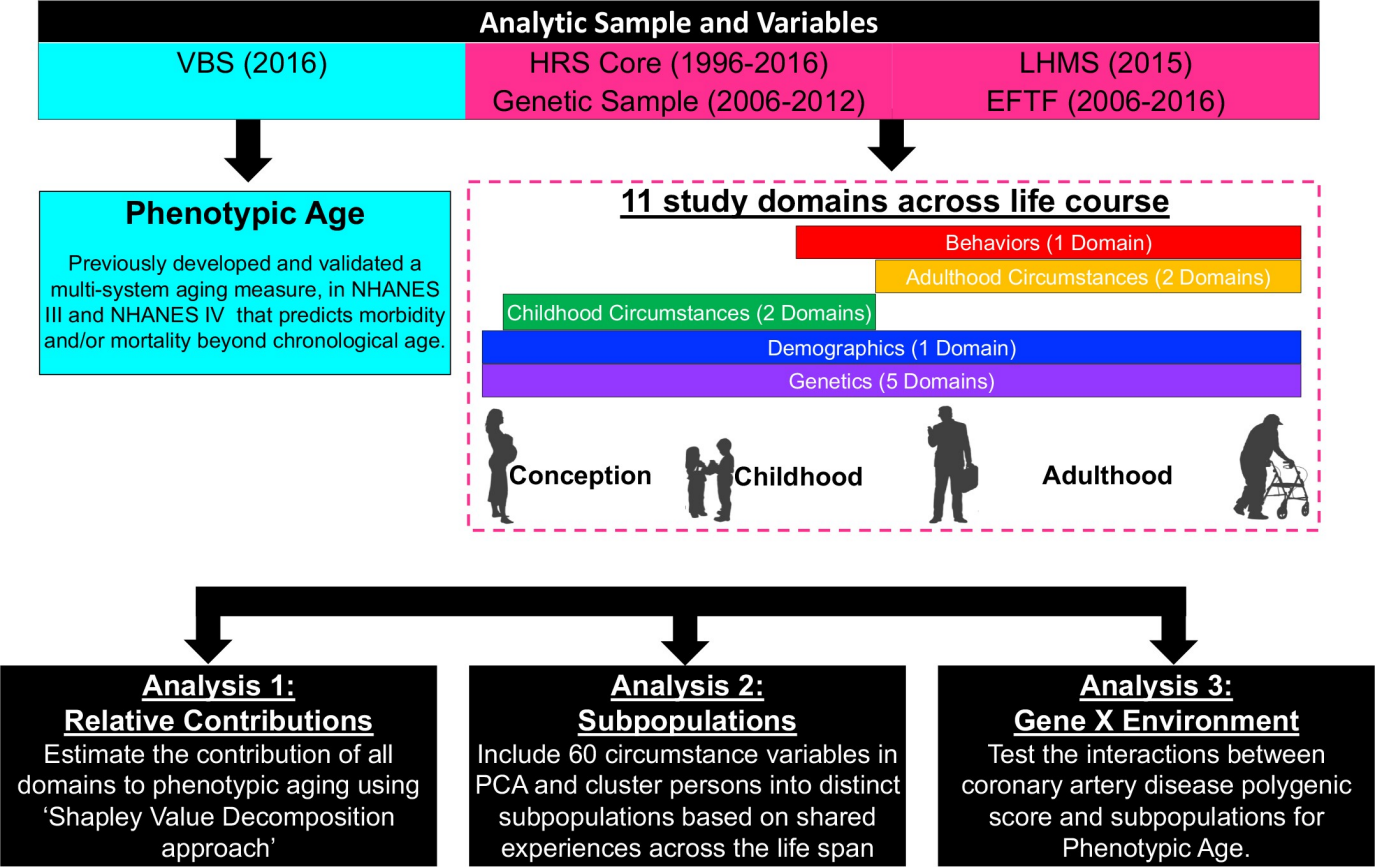
Roadmap for evaluating the association of genetics, behaviors, and life course circumstances with PhenoAge. The roadmap depicts our analytical procedures. We assembled analytic samples and a large array of variables from 4 components within HRS, including the core survey (1996–2016), the newly released 2015 LHMS, the EFTF interview (2006–2016), and the 2016 VBS. We also restricted the sample to participants who were part of the genetic sample (i.e., had a saliva sample collected in the EFTF interview [2006–2012]), leaving a final analytic sample of 2,339 persons. We categorized a large array of variables across the life course into 11 study domains, including 5 PGS domains (genetics), 2 childhood circumstances domains, 2 adulthood circumstances domains, 1 behavior domain, and 1 demographic domain. The relation between study domains and individual variables can be found in S1 Appendix and S1 Table. We then performed 3 analyses to evaluate the association of these domains (particularly the childhood and adulthood circumstances domains) with PhenoAge. EFTF, Enhanced Face-To-Face; HRS, Health and Retirement Study; LHMS, Life History Mail Survey; NHANES, National Health and Nutrition Examination Survey; PCA, principal component analysis; VBS, Venous Blood Study.

Since no consensus has been reached regarding the selection and definitions of variables characterizing childhood and adulthood circumstances, we considered a comprehensive set of factors suggested by existing literature to be associated with age-related health outcomes. All questions and corresponding responses/descriptions are provided in S1 Table. In brief, we defined 4 domains of childhood and adulthood circumstances: childhood SES, childhood adversity, adulthood SES, and adulthood adversity (Fig 2). These circumstance domains were also referred to as socioenvironmental circumstances. To enhance the clarity of our definitions, a full list of terminology (e.g., domains, circumstances) used in this study is provided in S1 Appendix.

### Behaviors

We included 1 domain for behaviors in this study, involving obesity, smoking, alcohol consumption, and physical activity. For obesity, we defined a variable called proportion of experiencing obesity (proportion obesity, range 0–1) as the percentage of survey waves for which a participant experienced a body mass index over 30 kg/m^2^. We used the most recent response to define smoking since this behavior largely did not change and the recent response would reflect a person’s recent health status. Three categories were defined for smoking: never smoking, former smoking, and current smoking. More details can be found in S1 Table and S1 Appendix.

### Genetic factors

To further differentiate the effect of socioenvironmental circumstances on phenotypic aging due to genetic predisposition (i.e., beyond one’s control), we included 5 domains for genetic factors based on previously established PGSs: anthropometrics, disease/longevity, mental health/personality, education/cognition, and smoking (details can be found in S1 Appendix). The saliva samples for genotyping SNPs were collected in the EFTF interviews from 2006 to 2012, and details on the construction of these PGSs are provided elsewhere [32].

### PhenoAge and phenotypic aging (PhenoAgeAccel)

PhenoAge was first developed and validated using independent waves from the National Health and Nutrition Examination Survey [4]. In brief, PhenoAge was derived from chronological age and 9 biomarkers, which included albumin, creatinine, glucose, (log) C-reactive protein, lymphocyte percent, mean cell volume, red cell distribution width, alkaline phosphatase, and white blood cell count. The score was calculated as a weighted (coefficients available in our previous publication [4]) linear combination of these variables, which was then transformed into units of years using 2 parametric (Gompertz distribution) proportional hazard models—one for the linearly combined score for all 10 variables and another for chronological age. Thus, PhenoAge represents the expected age within the population that corresponds to a person’s estimated hazard of mortality as a function of his/her biological profile [4,5].

Next, we calculated a measure, PhenoAge Acceleration (PhenoAgeAccel), defined as the residual resulting from a linear model when regressing PhenoAge on chronological age. Therefore, PhenoAgeAccel represents phenotypic aging after accounting for chronological age (i.e., whether a person’s biological profile is reflective of being older [positive value] or younger [negative value] than expected based on his/her chronological age).

### Statistical analyses

Fig 2 provides an overview of our analytical procedures; more complete details can be found in S1 Appendix. Briefly, in the first step, to evaluate the overall and relative contributions of all variables including childhood and adulthood circumstances, behaviors, and genetics to differences in PhenoAge after accounting for chronological age (which is essentially PhenoAgeAccel), we used the Shapley approach with mean logarithmic deviation. Compared with other decomposition methods, the Shapley approach has substantial advantages, such as being order independent (i.e., the order of circumstances for decomposition does not influence the results) and being able to sum components to produce the total value.

In the second step, to further evaluate the effect of childhood and adulthood circumstances on PhenoAge, we used a series of analyses. First, we performed a principal component analysis (PCA) for the 60 (mainly categorical) variables included in the 4 circumstance domains (childhood SES, childhood adversity, adulthood SES, and adulthood adversity). Second, we selected the top 4 principal components, based on the proportional variance explained, and used them as inputs in a hierarchical clustering analysis (HCA), in which we clustered participants into distinct subpopulations/clusters. These subpopulations can be taken to represent groups of participants with shared socioenvironmental circumstances. We then compared the PhenoAge of the subpopulations after accounting for chronological age to determine whether some subpopulations exhibited signs of accelerated aging. Finally, for every subpopulation/cluster, we estimated participants’ cluster membership (a continuous measure ranging from −1 to 1), which denotes how similar a participant’s profile is to the characteristics of the subpopulation/cluster. For instance, having a score of 0.8 for cluster 1 and −0.6 for cluster 2 suggests an individual is very similar to the profile represented by cluster 1, but not cluster 2. We then related these cluster membership values to the circumstances measures to determine which characteristics defined each subpopulation/cluster.

In the third step, we used multivariate models to examine the associations of PhenoAge with the subpopulations, behaviors, and individual PGSs (determined by assessing the magnitude of their associations with PhenoAge), with adjustment for covariates such as chronological age, sex, and ancestry (non-Hispanic white or non-Hispanic black). In these models, we also examined gene-by-environment interactions, by testing whether the PGSs and subpopulations, as well as behaviors, were related to PhenoAge in a multiplicative manner.

## Results

### The characteristics of the study population

As shown in Table 1, the average chronological age of the study population was 69.4 years (standard deviation [SD] = 11.1). About 56% (*n =* 1,392) were female, and the majority were non-Hispanic white (93.9%). There was a sizable proportion of participants reporting low childhood SES, e.g., 23.1% reported being financially poor in childhood. The prevalence varied for the 10 childhood traumatic events, from 0.8% (lived in orphanage) to 19.3% (parents abused drugs or alcohol). As adults, about 12% of participants reported receiving food stamps, 16% were not satisfied with their current financial situation, and over 20% found it at least somewhat difficult meeting payments on bills. The average education was 13.9 years (SD = 2.8). In general, variations in exposure to adulthood adversity were also found. For example, one-quarter reported having the experience of a spouse or child battling a life-threatening illness, while less than 4% of participants reported ever firing a weapon in combat.

**Table 1:** Characteristics of study population: Health and Retirement Study, *n =* 2,339.

Characteristic			Mean (SD) or *n* (%)
**Demographics**			
	Age, years		69.4 (11.1)
	Sex		
		Male	957 (44.2%)
		Female	1,392 (55.8%)
	Race/ethnicity		
		Non-Hispanic white	2,039 (93.9%)
		Non-Hispanic black	300 (6.1%)
**Childhood circumstances**			
	**Childhood SES**		
		Relocated due to financial difficulties	371 (15.5%)
		Family received financial help	341 (14.2%)
		Self-reported family poverty	
		Pretty well off financially	168 (8.2%)
		About average	1,579 (68.7%)
		Poor	592 (23.1%)
		Parental education, years	11.9 (3.7)
		Father lost jobs	
		No	1,730 (75.2%)
		Yes	461 (19.7%)
		Father never worked/always disabled	13 (0.5%)
		Never lived with father/father was not alive	135 (4.6%)
	**Childhood adversity**		
		**Childhood traumas**	
		Trouble with police	137 (6.5%)
		Repeated school	322 (12.9%)
		Physically abused	145 (6.2%)
		Parents used drugs or alcohol	439 (19.3%)
		Parents died	461 (17.5%)
		Separated from mother	237 (9.2%)
		Separated from father	474 (18.0%)
		Lived in orphanage	22 (0.8%)
		Lived in a foster home	27 (1.2%)
		Parents separated or divorced	301 (11.8%)
		**Childhood health**	
		Self-reported health	
		Excellent	1,324 (57.8%)
		Very good	588 (25.4%)
		Good	314 (12.5%)
		Fair	90 (3.6%)
		Poor	23 (0.7%)
		Disabled for 6 months	93 (4.3%)
		Head injury	248 (11.6%)
**Adulthood circumstances**			
	**Adulthood SES**		
		Education, years	13.9 (2.8)
		Ever received Medicaid	219 (7.1%)
		Ever received food stamps	325 (11.8%)
		Proportion of experiencing unemployment, 0–1	0.03 (0.1)
		Total wealth, dollars	
		Quintile 1	−5,438 (125,873)
		Quintile 2	58,185 (24,665)
		Quintile 3	151,192 (39,526)
		Quintile 4	303,823 (75,819)
		Quintile 5	1,153,861 (2,135,321)
		Satisfaction with present financial situation	
		Completely satisfied	468 (19.8%)
		Very satisfied	735 (32.6%)
		Somewhat satisfied	719 (31.0%)
		Not very satisfied	294 (11.8%)
		Not at all satisfied	123 (4.8%)
		Difficulties for meeting payments on bills	
		Not at all difficult	1,114 (48.3%)
		Not very difficult	672 (30.3%)
		Somewhat difficult	417 (16.4%)
		Very difficult	104 (3.8%)
		Completely difficult	32 (1.3%)
	**Adulthood adversity**		
		**Adulthood traumas**	
		Experienced the death of a child	327 (11.8%)
		Experienced a natural disaster	370 (16.1%)
		Fired a weapon in combat	86 (3.8%)
		Been a victim of a physical attack	152 (6.5%)
		Ever had life-threatening illness	561 (23.3%)
		Ever had a spouse or child with life-threatening illness	625 (24.8%)
		Spouse, partner, or child ever been addicted to drugs or alcohol	441 (18.4%)
		**Neighborhood physical disorder, range 1 (first statement) to 7 (second statement)**	
		There is no problem with vandalism and graffiti in this area/Vandalism and graffiti are a big problem in this area	2.2 (1.8)
		People feel safe walking alone in this area after dark/People would be afraid to walk alone in this area after dark	2.4 (1.9)
		This area is kept very clean/This area is always full of rubbish and litter	2.2 (1.7)
		There are no vacant or deserted houses or storefronts in this area/There are many vacant or deserted houses or storefronts in this area	2.4 (2.1)
		**Lifetime discrimination**	
		Unfairly dismissed from a job	488 (21.8%)
		Unfairly ever not been hired for a job	194 (8.8%)
		Ever been unfairly denied a promotion	233 (10.3%)
		Unfairly been prevented from moving into a neighborhood	49 (1.6%)
		Ever been unfairly denied a bank loan	113 (4.5%)
		Ever been unfairly treated by the police	133 (5.8%)
		**Chronic stressors**	
		Ongoing health problems (in yourself)	
		No, didn’t happen	709 (32.4%)
		Yes, but not upsetting	759 (31.0%)
		Yes, somewhat upsetting	685 (28.8%)
		Yes, very upsetting	186 (7.8%)
		Ongoing physical or emotional problems (in spouse or child)	
		No, didn’t happen	1276 (56.0%)
		Yes, but not upsetting	374 (15.1%)
		Yes, somewhat upsetting	510 (21.1%)
		Yes, very upsetting	179 (7.7%)
		Ongoing problems with alcohol or drug use in family member	
		No, didn’t happen	1894 (80.9%)
		Yes, but not upsetting	142 (6.1%)
		Yes, somewhat upsetting	209 (8.9%)
		Yes, very upsetting	94 (4.1%)
		Ongoing difficulties at work	
		No, didn’t happen	1951 (80.7%)
		Yes, but not upsetting	219 (10.8%)
		Yes, somewhat upsetting	142 (7.5%)
		Yes, very upsetting	27 (1.2%)
		Ongoing financial strain	
		No, didn’t happen	1392 (59.9%)
		Yes, but not upsetting	484 (20.4%)
		Yes, somewhat upsetting	362 (15.4%)
		Yes, very upsetting	101 (4.3%)
		Ongoing housing problems	
		No, didn’t happen	1968 (84.9%)
		Yes, but not upsetting	223 (9.1%)
		Yes, somewhat upsetting	118 (4.6%)
		Yes, very upsetting	30 (1.4%)
		Ongoing problems in a close relationship	
		No, didn’t happen	1779 (75.5%)
		Yes, but not upsetting	279 (11.9%)
		Yes, somewhat upsetting	224 (9.7%)
		Yes, very upsetting	57 (2.7%)
		Helping at least 1 sick, limited, or frail family member or friend on a regular basis	
		No, didn’t happen	1506 (63.9%)
		Yes, but not upsetting	531 (22.9%)
		Yes, somewhat upsetting	240 (10.6%)
		Yes, very upsetting	62 (2.6%)
		**Life events**	
		Involuntarily lost a job for reasons other than retirement	213 (11.1%)
		Unemployed	210 (9.9%)
		Anyone else in your household unemployed	261 (11.5%)
		Moved to a worse residence or neighborhood	43 (2.5%)
		Robbed or burglarized	135 (5.9%)
		**Major events**	
		Ever been in a jail	68 (2.6%)
		Ever been in a hospital	114 (4.5%)
		Ever lived in a combat zone	118 (5.4%)
		Ever lived in military housing	499 (21.2%)
		Ever been homeless	47 (2.0%)
**Behaviors**			
		Proportion of experiencing obesity, 0–1	0.3 (0.5)
		**Smoking**	
		Never smoking	1115 (47.3%)
		Former smoking	1037 (44.4%)
		Current smoking	187 (8.3%)
		**Alcohol consumption**	
		Ever drinking	1377 (63.1%)
		Drinking days per week	1.5 (2.7)
		Number of drinks per drinking day	0.9 (1.8)
		**Activities**	
		Vigorous activities age 18–29 years	734 (35.9%)
		Vigorous activities age 30–39 years	591 (29.1%)
		Vigorous activities age 40–49 years	539 (26.2%)
		Moderate activities age 18–29 years	1,078 (49.3%)
		Moderate activities age 30–39 years	1,015 (46.8%)
		Moderate activities age 40–49 years	990 (45.1%)

Percentages (%) were weighted. The percentages may not sum to 100 because of rounding. As described in the Methods, we defined the proportion of experiencing obesity (the proportion of observations that met criteria for obesity, range 0–1) as the percentage of survey waves for which a participant had a measured BMI over 30 kg/m^2^.

SD, standard deviation; SES, socioeconomic status.

Obesity had a prevalence of about 30% over the entire study period. About 44% of participants were former smokers, and 8% were current smokers. Over 60% of participants consumed alcohol, and on average participants drank 1.5 times per week, and averaged about 1 drink on those days. Engaging in vigorous and/or moderate physical activity was most common during young adulthood and subsequently deceased as participants aged. Finally, the average level of PhenoAgeAccel was –0.38, with 927 participants having accelerated phenotypic aging (i.e., positive value) and 1,412 having decelerated phenotypic aging (i.e., negative value). This suggests that our analytical sample is slightly healthier than the general HRS sample, given that PhenoAgeAccel was estimated in the full sample, and by definition had a mean of 0.

### Potential contributions to phenotypic aging

**Fig 3:**
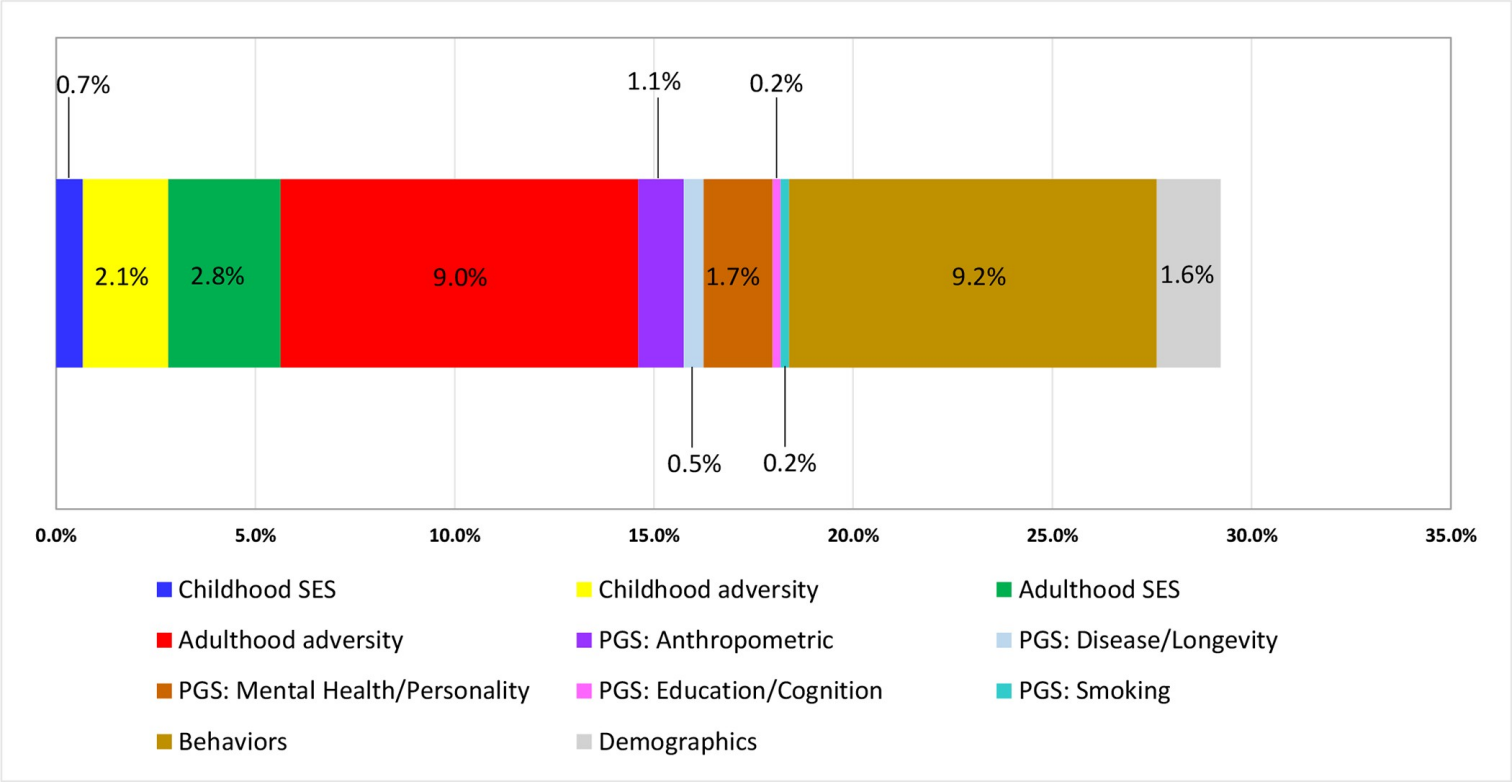
The contribution of all 11 study domains to PhenoAgeAccel. The 11 domains include 4 childhood and adulthood circumstances domains, 5 PGS domains, 1 behavior domain, and 1 demographic domains. Overall, the 11 study domains contributed 29.2% (bootstrap standard error = 0.003) of the variance in PhenoAgeAccel. PGS, polygenic score; SES, socioeconomic status.

Fig 3 presents the results from the Shapley approach, depicting the proportions of the variance explained by all 11 study domains. Collectively, the factors accounted for 29.2% (bootstrap standard error [SE] = 0.003) of the variance in PhenoAgeAccel. Overall, behaviors had the largest single contribution to PhenoAgeAccel (9.2%, bootstrap SE = 0.004). Among the 4 childhood and adulthood circumstances domains, adulthood adversity was the largest contributor (9.0%, bootstrap SE = 0.003), while adulthood SES, childhood adversity, and childhood SES accounted for 2.8% (bootstrap SE = 0.002), 2.1% (bootstrap SE = 0.003), and 0.7% (bootstrap SE = 0.001), respectively. All 5 domains of PGSs contributed 3.8% of the variance in PhenoAgeAccel, with the largest proportion coming from the PGS domain of mental health/personality (1.7%, bootstrap SE = 0.002), followed by anthropometric measures (1.1%, bootstrap SE = 0.002), disease/longevity (0.5%, bootstrap SE = 0.001), education/cognition (0.2%, bootstrap SE = 0.001), and smoking (0.2%, bootstrap SE = 0.001).

### Profiles of childhood and adulthood circumstances and their relation to phenotypic aging

**Fig 4:**
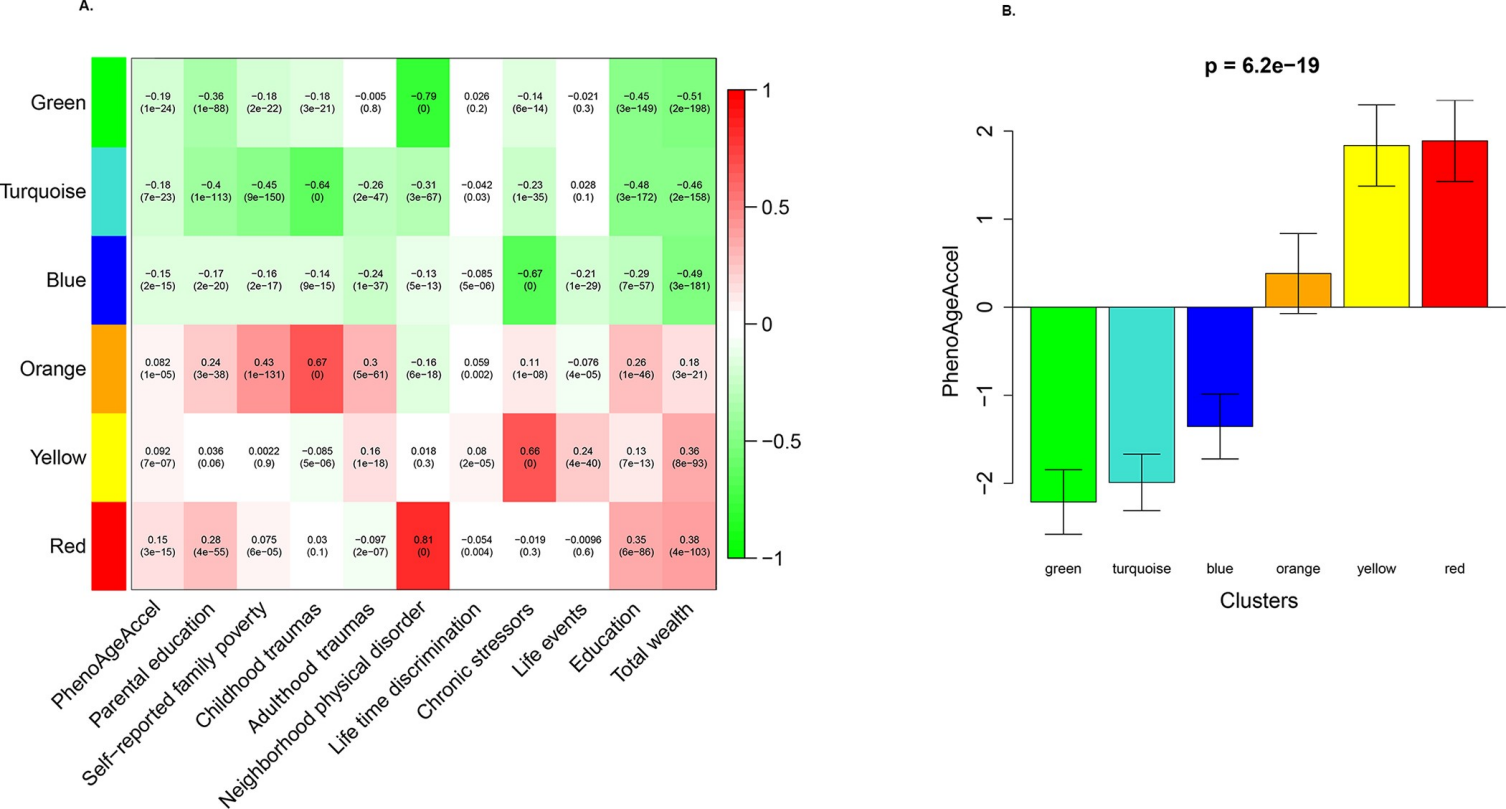
Cluster membership–trait correlations and PhenoAgeAccel across 6 clusters. (A) Cluster membership–trait correlations and *p*-values; (B) PhenoAgeAccel across the 6 clusters. In (A), to determine what each subpopulation/cluster represents, we calculated a continuous measure (cluster membership) for each cluster (between −1 and 1) that denotes how strongly a person belongs to that given cluster—for instance, someone may have a score of 0.8 for the green cluster and −0.6 for the red cluster, suggesting that he/she is very similar to the profile represented by the green cluster, but not the red cluster. Each cell reports the correlation (and *p*-value) resulting from correlating cluster membership (rows) to traits (columns, including PhenoAgeAccel and summarized measures of several circumstances). The table is color-coded by correlation according to the color legend. PhenoAgeAccel, Phenotypic Age Acceleration.

Using input from 4 principal components derived using 60 variables, HCA was used to cluster individuals into subpopulations. Overall, we identified 6 distinct subpopulations characterized by shared childhood and adulthood circumstances (S1 Fig, top colored row). Those assigned to the red subpopulation in Fig 4A are characterized by having lower levels of education, having poor financial situations during childhood and adulthood, having lower educated parents, and living in neighborhoods with severe physical disorder. Conversely, the green subpopulation includes participants who were more likely to have higher adult SES, to have had moderately higher childhood SES, and to live in neighborhoods with lower levels of physical disorder. The turquoise subpopulation includes participants with the highest SES in childhood and adulthood, but whose neighborhoods are slightly more disordered than those in the green cluster. Those in the yellow subpopulation are mainly characterized by having higher levels of chronic stress, while those in the orange subpopulation had low SES in childhood and higher levels of childhood trauma. Finally, the blue subpopulation represents participants with moderate SES in childhood, and much lower levels of chronic stress than other clusters. S2 Fig shows the correlation among the 6 subpopulations. An inverse association was observed between the green and red subpopulations—suggesting that participants assigned to them have opposite life course circumstances. Similarly, the turquoise and orange subpopulations appeared to have opposite circumstances, as did the yellow and blue subpopulations.

Bivariate differences in PhenoAgeAccel between the 6 subpopulations are presented in Fig 4B (see S3 Fig for a violin plot). Two of these subpopulations (i.e., red and yellow) stood out as disadvantaged, exhibiting significantly higher PhenoAges on average. More specifically, participants assigned to the red or yellow subpopulations were about 1.75 years older phenotypically than expected based on their chronological ages. Conversely, advantaged subpopulations, such as those in the green or turquoise subpopulations, had PhenoAges that were about 2 years younger than expected, while those in the blue subpopulation were a little over 1 year younger than expected.

### Gene-by-environmental interactions

**Fig 5:**
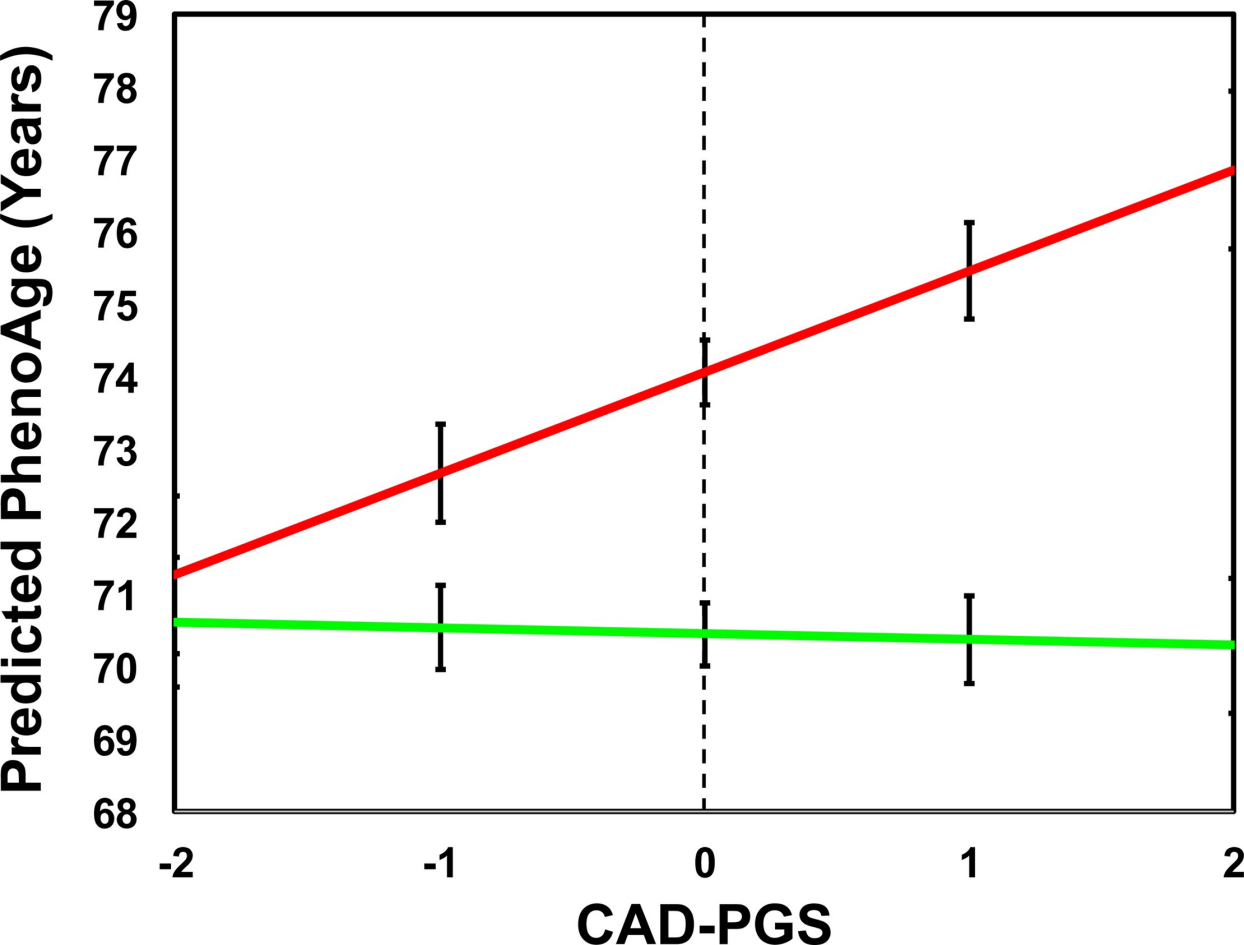
The significant interaction between CAD-PGS and the red (relative to the green) subpopulation for PhenoAge. This figure is based on results from the multivariate models in Table 2, with adjustment for chronological age, sex, ancestry, proportion obesity, smoking, and the CAD-PGS × cluster interaction terms. We provide an example of 2 predictions, one for those in the red subpopulation/cluster and another for those in the green subpopulation/cluster (setting all other confounding variables to the mean). Therefore, both groups are equivalent on all variables, and the only things that differ are the main effects for the clusters and the interaction effect of CAD-PGS (e.g., 2 SDs below the population mean). CAD-PGS, polygenic score for coronary artery disease; PhenoAge, Phenotypic Age.

Because a PGS for coronary artery disease (CAD-PGS) (in the disease/longevity domain) accounted for the highest proportion of variance in PhenoAge of all the PGSs (S4 Fig), we tested for main effects and gene-by-environment interactions for CAD-PGS, the 6 environmental clusters, and 2 important behavioral variables: smoking and obesity. Relative to the green subpopulation, those in the red, yellow, and orange subpopulations had significantly higher PhenoAges (Table 2). For instance, in a fully adjusted main-effect model, those assigned to the red subpopulation on average had PhenoAges that were more than 3.5 years older than those assigned to the green subpopulation (β = 3.60, *p* < 0.001). We also observed a significant main effect for the CAD-PGS, such that every 1-SD increase in the PGS was associated with a 0.44-year increase in PhenoAge (β = 0.44, *p* = 0.014). Both obesity and smoking were also associated with higher PhenoAges—each 1-SD increase in the proportion of observations a participant qualified as obese (BMI > 30 kg/m^2^), was associated with a nearly 2.5-year increase in PhenoAge (β = 2.44, *p* < 0.001), while current smokers on average had PhenoAges that were 3.6 years older than never smokers (β = 3.55, *p* < 0.001). When considering gene-by-environment interactions, we observed a significant interaction between CAD-PGS and the red (relative to the green) subpopulation (β = 1.48, *p* = 0.020). This being said, while on average those in the red subpopulation had PhenoAges that were 3.6 years higher than those in the green subpopulation, the differences were exacerbated among individuals who also were genetically predisposed to coronary artery disease (CAD), suggesting that such an environment is even more harmful for those with increased genetic vulnerability to CAD. As illustrated in Fig 5, a higher CAD-PGS increased the difference in PhenoAge between those in the red versus the green subpopulation in a multiplicative manner, such that among participants with a CAD-PGS that was 2 SDs below the mean, those in the red subpopulation had slightly higher average PhenoAge (+0.6 years) than those in the green subpopulation. However, among those with a CAD-PGS that was 2 SDs above the mean, those in the red subpopulation had substantially higher average PhenoAge (+6.6 years) than those in the green subpopulation.

**Table 2:** Associations of PhenoAge with CAD-PGS, behaviors, and childhood and adulthood circumstances.

Trait or interaction	Model 1		Model 2		Model 3		Model 4	
	Coef. (SE)	*p-*Value	Coef. (SE)	*p-*Value	Coef. (SE)	*p-*Value	Coef. (SE)	*p-*Value
**6 subpopulations/clusters for childhood and adulthood circumstances**								
Green	Ref.		Ref.		Ref.		Ref.	
Turquoise	0.82 (0.58)	0.158	0.83 (0.58)	0.152	0.85 (0.58)	0.143	0.79 (0.58)	0.169
Blue	−0.06 (0.61)	0.922	−0.05 (0.61)	0.939	−0.06 (0.61)	0.926	−0.08 (0.61)	0.899
Orange	1.91 (0.66)	0.004	1.94 (0.66)	0.003	1.88 (0.66)	0.004	1.93 (0.66)	0.003
Yellow	3.42 (0.61)	<0.001	3.46 (0.61)	<0.001	3.44 (0.61)	<0.001	3.42 (0.61)	<0.001
Red	3.60 (0.63)	<0.001	3.61 (0.63)	<0.001	3.57 (0.63)	<0.001	3.59 (0.63)	<0.001
**Proportion obesity**	2.44 (0.18)	<0.001	2.43 (0.18)	<0.001	2.45 (0.18)	<0.001	2.44 (0.18)	<0.001
**Smoking**								
Never smoking	Ref.							
Former smoking	1.36 (0.37)	<0.001	1.38 (0.37)	<0.001	1.38 (0.37)	<0.001	1.35 (0.37)	<0.001
Current smoking	3.55 (0.68)	<0.001	3.53 (0.68)	<0.001	3.56 (0.68)	<0.001	3.61 (0.68)	<0.001
**CAD-PGS**	0.44 (0.18)	0.014	−0.08 (0.40)	0.841	0.44 (0.18)	0.013	0.55 (0.26)	0.032
**Gene-by-environmental interactions**								
CAD-PGS × green			Ref.					
CAD-PGS × turquoise			0.44 (0.56)	0.431				
CAD-PGS × blue			0.52 (0.58)	0.371				
CAD-PGS × orange			0.64 (0.66)	0.332				
CAD-PGS × yellow			0.40 (0.60)	0.505				
CAD-PGS × red			1.48 (0.64)	0.020				
CAD-PGS × proportion obesity					0.45 (0.18)	0.012		
CAD-PGS × never smoking							Ref.	
CAD-PGS × former smoking							−0.07 (0.37)	0.845
CAD-PGS × current smoking							−0.96 (0.66)	0.147

As described in Methods, we defined the proportion of experiencing obesity (proportion obesity; range 0–1) as the percentage of survey waves for which a participant experienced a BMI over 30 kg/m^2^. The numbers of participants in each cluster were *n =* 438 for green, *n =* 593 for turquoise, *n =* 470 for blue, *n =* 392 for orange, *n =* 503 for yellow, and *n =* 460 for red. Model 1 adjusted for chronological age, sex, ancestry, proportion obesity, and smoking. Model 2, 3, and 4 additionally added the CAD-PGS × cluster interaction terms, the CAD-PGS × proportion obesity interaction term, and the CAD-PGS × smoking interaction term, respectively.

BMI, body mass index; CAD-PGS, polygenic score for coronary artery disease; Coef., coefficient; PhenoAge, Phenotypic Age; SE, standard error.

In addition to PGS-by-subpopulation interactions, we also observed a significant interaction between CAD-PGS and proportion obesity (proportion of experiencing obesity). This suggests that the increase in PhenoAge accompanying every 1-SD increase in the obesity measure was multiplicatively increased by 0.45 years with every 1-SD increase in the CAD-PGS. Finally, no interactions were observed between smoking and CAD-PGS.

## Discussion

Based on data from a large sample of older adults in the US, our comprehensive analyses showed that childhood and adulthood circumstances, behaviors, and genetic factors were associated with differences in a novel multi-system signature of aging. Collectively, the factors we evaluated accounted for just under one-third of the variance in phenotypic aging. Using many of the variables characterizing childhood and adulthood circumstances, we were able to group participants into 6 subpopulations—2 of which appeared to reflect disadvantaged subpopulations exhibiting substantially increased phenotypic aging. Finally, we observed a significant gene-by-environment interaction between a previously validated PGS for CAD and the seemingly most disadvantaged subpopulation, suggesting a multiplicative effect of adverse life course circumstances coupled with genetic risk on phenotypic aging. Taken together, these results may inform potential interventions to reduce morbidity and mortality risk experienced throughout the life course. While causality needs to be formally evaluated, the results from the current study highlight the socioenvironmental circumstances and behavioral factors that potentially have the largest influence over level of phenotypic aging. As such, targeting these factors may lead to improvements in health and diminish corresponding disparities over the life course.

Results from both the Shapley approach and HCA draw attention to adversity in adulthood as a potential driver of differences in phenotypic aging, i.e., subsequent disparities in morbidity and mortality risk. More specifically, persons with higher levels of neighborhood physical disorder or high chronic stress—which were the defining characteristics of the red and the yellow subpopulations/clusters—appear to be phenotypically older than their peers. Our study extends results from earlier studies reporting that persons living in neighborhoods characterized by poor environments (e.g., lower aesthetic quality and safety) exhibited accelerated cellular aging, as denoted by shorter telomere length [33,34]. Poor neighborhood environments may induce stress, predispose one to stressful life events, or shape exposure and vulnerability to stress [35]. To date, both human and animal research have documented the negative effects of stress on health and aging [36,37]. For instance, the literature on “allostatic load” postulates that stressful life experiences may contribute to multi-system dysregulation. However, by considering multiple potential stressors simultaneously using advanced statistical approaches, we were able to assess their relative contributions. Our results suggest that preventing or reducing these adversities (e.g., through improvements in neighborhood safety and increasing affordability of housing) should be prioritized in efforts to improve population health, particularly in the face of rapid population aging in the US and worldwide.

Similarly, our results add further evidence to substantiate the link between adulthood SES and healthy aging. Education is thought to act as a robust indicator of SES, contributing to social gradients. In this study, the characteristic that best defined the 3 advantaged subpopulations (i.e., green, turquoise, and blue) was higher levels of education (Fig 4A). This is in stark contrast to the 3 disadvantaged clusters—all of which were characterized by low education. Chronic socioeconomic deprivation associated with low education is thought to provoke a number of adverse biological responses, including a gene expression profile called the conserved transcriptional response to adversity, characterized by increased proinflammatory signaling, and downregulation of antiviral type I interferon and antibody-related genes [38]. Over time, this transcriptional profile may increase susceptibility to a number of age-related chronic diseases, possibly through acceleration of the biological aging process, although the causal relationship between these transcriptional changes and our measures of biological aging remains unclear.

As with adulthood SES, a large number of studies have highlighted the influence of childhood circumstances (e.g., SES and adversity) [10–17]. For instance, Hayward and Gorman suggested that childhood social and economic conditions get embedded within one’s biology and have far-reaching implications for one’s health as one ages [39]. However, it is important to account for the fact that individuals who experience disadvantages in childhood are more likely to go on to experience adverse circumstances in adulthood, which could lead to overestimation of the effect of childhood circumstances in traditional regression analysis. In this study, we applied the Shapley approach to appropriately decompose the contributions of childhood and adulthood circumstances, providing relatively accurate estimates compared to those from traditional methods. We observed an influence of childhood SES and adversity on phenotypic aging, suggesting that adversity in childhood may influence aging beyond predisposing a person to adversity in adulthood.

Unlike socioenvironmental circumstances, which are mostly beyond the individual’s control, health behaviors are much more modifiable and, more importantly, appear to have the greatest contribution to the variance in phenotypic aging in this study (9.2%). This finding highlights the importance of strategies aimed at attenuating modifiable risk factors like obesity and smoking. A recent observational study showed that the American population today is experiencing “delayed aging” and better health compared to the population during the late 1980s. While this can be partially explained by changes in smoking habits and increased use of antihypertensive and cholesterol-lowering medications, these changes are being counteracted by exorbitantly high obesity rates [40]. Generally, rising BMI in the US has stunted potential gains in life expectancy—such as those as experienced by other high-income countries [30]. To inform policy, research is needed to assess the effectiveness of behavioral interventions on measures of aging [41]. If the health behaviors considered in this analysis are shown to be causal drivers of phenotypic aging, they represent a targetable strategy for reducing disease burden. While interventions to promote exercise, smoking and/or drinking cessation, and weight reduction are not new to public health, measures like PhenoAge provide a useful intermediate phenotype that can be used to either target interventions for at-risk groups and/or track progress and intervention efficacy. From an individual standpoint, a more immediate measure like PhenoAge could be a more powerful and concrete motivator than are future risk assessments.

While our results highlight the influence of a variety of factors on phenotypic aging, the detrimental effects were not consistent across individuals. Our results suggest a moderating effect of genetic predisposition, such that individuals who have an innate susceptibility to diseases, such as CAD, may suffer even more from experiences of chronic stress and adversity, and/or the negative implications of obesity. For instance, among participants with low genetic risk for CAD, we showed that the differences in PhenoAge between those in the most adverse social environments (red cluster) and those in the most advantageous (green cluster) were minimal. However, among individuals with high genetic risk for CAD, we observed much higher PhenoAges for those in the red relative to the green cluster—suggesting that genetic predisposition may multiply the impact of social environment in a super-additive synergistic manner. Similar results were also observed for the obesity-by-CAD-PGS interaction, suggesting that genetic predisposition may also compound the association between extreme adiposity and aging. While there are previous reports on interactions between genes (PGSs and specific genes such as APOE ε4) and environmental factors (e.g., SES, education, exercise, and stressful life events) for aging-related outcomes (e.g., cognition and self-reported health) (e.g., [42–47]), no studies to our knowledge have examined the interaction between PGSs and a comprehensive set of life course circumstances for a single aging measure like PhenoAge. Our gene-by-environment results are noteworthy, given that we have previously shown that every 1-year increase in PhenoAge, relative to chronological age, is associated with a 9% increased risk of dying [5]. This finding is particularly concerning given that the red subpopulation (relative to the green) was associated with a 3.6-year increase in PhenoAge, each 1-SD increase in CAD-PGS was associated with about a half year increase in PhenoAge, and, together, they had a multiplicative effect. Based on these findings, targeting interventions and policies towards disproportionally at-risk individuals may have significant public health implications.

Despite the availability of a comprehensive set of life course circumstances and the innovative application of interdisciplinary approaches (e.g., the Shapley approach), the results should be interpreted with caution. First, efforts to assemble a comprehensive set of circumstances from several components of HRS reduced sample size and potentially altered the population structure. For instance, a sizable proportion of persons who attended the 2016 VBS were excluded due to missing data on childhood adversity, collected through the 2015 LHMS. Furthermore, PGSs were only available for persons of European or African ancestry. As a result, we observed slight differences in relevant characteristics of our analytic sample compared with those excluded from the analyses. This issue was slightly offset by using dummy variables for missingness in order to retain participants and by considering other versions of survey weights in the sensitivity analyses—all of which produced findings consistent with those presented. However, as mentioned before, the representativeness of the study population is still problematic. Second, information on the specific timing of childhood and adulthood circumstances was not available. Previous studies have suggested that the earlier adversity develops, the greater the negative effect on health in later life [48]. Third, most of these circumstances were based on self-reports, and this retrospective approach leads to possible recall biases. Fourth, since we were unable to observe all childhood and adulthood SES and adversity factors, this type of decomposition tends to offer a more conservative estimate (e.g., underestimate) of their contribution to phenotypic aging. This possible underestimation also applies to genetics, as all the PGSs are merely a small set among a potentially large set of genetic factors that matter. Finally, we acknowledge that many unmeasured confounding factors may exist but were not accounted for in this study. Further, this study was not able to address questions of causality. In future research, it will be important to examine associations between childhood adversity/SES and measures such as PhenoAge longitudinally and at earlier life stages, in order to draw causal inferences, as well as track differences in rates of aging, rather than levels.

In conclusion, in a sample of US older adults, we estimated that genetic, behavioral, and socioenvironmental circumstances during both childhood and adulthood accounted for about 30% of the variance in phenotypic aging, i.e., difference in morbidity and mortality risk. Individually, both behaviors and socioenvironmental circumstances contributed to substantial amounts of the variance in phenotypic age. Furthermore, we identified disadvantaged subpopulations that exhibited accelerated aging and for whom genetic predisposition—in this case genetic risk for CAD—multiplied the associated increase in phenotypic aging. These findings highlight the influence of behaviors, socioenvironmental circumstances, and genetic predisposition for CAD on aging and health, and thus may directly inform policies surrounding targeted interventions and allocation of resources for promoting healthy aging, and ameliorating health disparities. While this study is unique in terms of the comprehensive set of variables and the outcome measure—PhenoAge—and may only reflect results specific to the US population, further validation of these findings is needed.

## Supporting information

S1 STROBE checklist (DOC)

S1 Appendix Methods and results. (DOCX)

S1 Fig The cluster dendrogram. Six subpopulations/clusters were identified, labeled “blue” (*n =* 470), “green” (*n =* 438), “turquoise” (*n =* 593), “orange” (*n =* 392), “yellow” (*n =* 503), and “red” (*n =* 460). For the categories of summarized measures given in the lower half of the figure, the closer to “dark orange,” the higher the dose of exposing to this risk factor (except race/ethnicity). For example, parental education more than 16 years was indicated by “dark magenta,” whereas parental education less than 12 years was indicated by “dark orange.”
(PDF)

S2 Fig Correlations among the cluster memberships for the 6 subpopulations/clusters. To determine what each group represents, we calculated a continuous measure (cluster membership) for each cluster (between −1 and 1) that denotes how strongly a person belongs to that given cluster—for instance, someone may have a score of 0.8 for the green cluster and −0.6 for the red cluster, suggesting he/she is very similar to the profile represented by the green cluster, but not that by the red cluster. Each cell reports the correlation (and *p*-value) between cluster memberships.
(PDF)

S3 Fig PhenoAgeAccel across 6 clusters (violin plot). (PDF)

S4 Fig *R*^2^ for PhenoAge explained by each PGS for multiple phenotypes after adjustment for chronological age, sex, proportion of experiencing obesity, smoking, and ancestry. (TIF)

S5 Fig The contribution of all 11 domains to PhenoAgeAccel in sample of individuals who fasted (*n =* 1,579). Overall, all the 11 domains contributed 31.7% (bootstrap standard error = 0.004) of variance in PhenoAgeAccel.
(PDF)

S6 Fig PhenoAgeAccel across 6 clusters in sample of individuals who fasted. (PDF)

S1 Table Questions and responses for variables included in the childhood and adulthood circumstances and behaviors. (DOCX)

## Abbreviations

BMI: body mass index
CAD: coronary artery disease
CAD-PGS: polygenic score for coronary artery disease
EFTF: Enhanced Face-To-Face
HCA: hierarchical clustering analysis
HRS: Health and Retirement Study
LHMS: Life History Mail Survey
PCA: principal component analysis
PGS: polygenic score
PhenoAge: Phenotypic Age
PhenoAgeAccel: Phenotypic Age Acceleration
SD: standard deviation
SE: standard error
SES: socioeconomic status
VBS: Venous Blood Study
