# Supplementary material for: Associations of genetics, behaviors, and life course circumstances with a novel aging and healthspan measure: Evidence from the Health and Retirement Study
Source: PLoS Med. 2019 Jun 18;16(6):e1002827. doi: 10.1371/journal.pmed.1002827 (PMC6581243; doi:10.1371/journal.pmed.1002827)
Supplement: S1 Appendix — (DOCX) [file pmed.1002827.s002.docx]

**S1 Appendix. Methods and results**

# **Methods**

## **Terminologies used in this study**

To enhance the clarity, we provided explanations for the terminologies used in this study as shown below. Note that all the explanations were based on this study and may not be suitable to other studies.

| **Terminology** | **Explanations** |
| --- | --- |
| Socioenvironmental circumstances | Childhood and adulthood variables, including socioeconomic status (SES), adversity, etc. |
| Childhood circumstances | All exposures/variables relevant to SES, adversity, etc. in childhood. |
| Adulthood circumstances | All exposures/variables relevant to SES, adversity, etc. in adulthood. |
| Domain | A group of variables/exposures.  Example: Childhood SES domain in this study |
| SES domain | A domain including all exposures/variables related to SES  Example: Childhood SES in this study |
| Adversity domain | A domain including all exposures/variables related to adverse experience, including traumas, health, stressors, etc.  Example: Childhood adversity in this study |
| Polygenic score (PGS) | Also called a polygenic risk score or genetic risk score, is a value based on variation in multiple genetic loci and their associated weights. It serves as the best prediction for the trait that can be made when taking into account variation in multiple genetic variants.  Example: “PGS for height” in this study |
| PhenoAge Acceleration (PhenoAgeAccel) | The residual resulting from a linear model when regressing PhenoAge on chronological age. Therefore, PhenoAgeAccel represents phenotypic aging after accounting for chronological age. |
| Shapley value decomposition | The idea of this decomposition is to understand not only how much all (childhood and adulthood) circumstances affect health inequality, but which specific circumstance contributes by how much to total inequality. |
| Principal component analysis (PCA) | A statistical method to reduce the dimensionality of potential correlated variables. It converts possibly correlated variables into a set of values of linearly uncorrelated variables named principal components (PCs), which can be used in further analysis. |
| Hierarchical clustering analysis (HCA) | A method of cluster analysis which aims to categorize similar objects (variables or participants) into subgroups/clusters. The endpoint is a set of clusters, where each cluster is distinct from each other cluster, and the objects within each cluster are broadly similar to each other. |
| Cluster membership | A concept used in WGCNA, denotes how similar a participant’s profile is to the characteristics represented by the cluster. It is estimated as the correlation between the participant’s scores across the variables used for clustering, and the first PC when only considering participants assigned to the cluster. It ranges from -1 to 1. For instance, someone may have a score of 0.8 for cluster 1 and -0.6 for cluster 2, suggesting s/he is very similar to the profile representative of cluster 1, but not cluster 2. |

## **Data**

## ***Component 1. The HRS Core Survey***

The HRS study has interviews every 2 years for collecting a wide range of information, including economics, health, marital, family status, and public and private support systems from 1992 to 2016 (the most recent wave) [1]. Although HRS has grown with additions of new cohorts, the contents of the core survey have generally remained consistent. For instance, the 2010 HRS core survey included multiple sections such as demographics and background, health, cognition, family structure and transfers, functional limitations, housing, physical measures, employment and pensions, disability, health services and insurance, expectations, assets and income, assets change, widowhood and divorce, wills, insurance, and trusts. In addition, HRS has experimental modules on specialized topics as part of the core survey. These modules only target a random subsample at the end of the core survey. For the present study, some variables in childhood SES were assembled from the section of health in the core survey and/or modules. Since RAND HRS groups have created user-friendly files with cleaned and processed variables with consistent and intuitive naming conventions, and model-based imputations, we assembled most of childhood SES variables from the RAND HRS files. Otherwise, we assembled variables from the original HRS released core data from 1996 (when the variables were available) to 2016.

***Component 2. The 2015 Life History Mail Survey***

The 2015 HRS Life History Mail Survey (LHMS) was conducted from December 2015 to August 2016 in a subsample of HRS participants (n=11,256). It aimed to collect information on residential history, education history, and other important childhood and family events. The target subsample included all living HRS participants who were not included in the 2015 Consumption and Activities Mail Survey (CAMS) and who completed their most recent HRS core survey interview in English (rather than Spanish). 6,481 participants were enrolled in the LHMS with a response rate of 58%. We assembled many variables for childhood traumas, major events in adulthood, and behaviors from the 2015 LHMS.

***Component 3. The Enhanced Face-to-Face Interview***

In 2006, HRS initiated the Enhanced Face-to-Face (EFTF) interview with a mixed-mode design during the follow-up period in which a random half of HRS participants were assigned a face-to-face interview with physical and biological measures (e.g., saliva sample) and a psychosocial questionnaire. The other half completed only the core survey but was selected for the next (i.e. 2008) EFTF interview. Similar method was applied to the subsequent HRS survey. In fact, the psychosocial questionnaire was piloted in 2004 and included personal evaluations of their life circumstances, subjective well-being, lifestyle and stress. Participants completed it and returned by mail. Most of life circumstances variables in the present study were assembled from this psychosocial questionnaire from 2006 to 2016. The saliva sample was used for genetic research after genotyping SNPs.

***Component 4. The 2016 Venous Blood Study***

To provide a substantially fuller picture of the health of the representative sample of older Americans, HRS began to collect venous blood for the first time in 2016. All panel participants in the core survey were asked to consent to attend the Venous Blood Study (VBS) except the proxy respondents and nursing home residents. This 2016 VBS was conducted in a separate home visit by a trained phlebotomist, from February 2016 to September 2017. The blood collection was managed by Hooper Holmes Health & Wellness. They contacted the consenting participants and scheduled the blood draw within 4 weeks of the HRS core survey. The details of the 2016 VBS 2016 data was provided in other reports [2]. The final sample size was 9,934, with a consent rate of 78.5% and a final completion rate of 65%. These biomarkers used to create PhenoAge in this study were from the 2016 VBS.

## **Childhood and adulthood circumstances**

## Although all questions and corresponding responses/descriptions for childhood and adulthood circumstances had been provided in Table S1, we described some definitions below.

***Childhood SES:***

We included four relevant variables that have been used before [3, 4], including relocated due to financial difficulties, family received financial help, self-reported family poverty, and highest years of education for father or mother. One additional variable—“father lose job”, indicating that the participants’ father experienced a significant unemployment spell (“several months or more”), was included, as it was suggested to appropriately reflect childhood SES [5]. This variable may incorporate information on family structure since it was assigned a value of 3 if the participant never lived with father or his/her father was not alive before the age of 16 [5].

***Childhood adversity:***

Childhood traumas: Due to the availability of data from the 2015 LHMS, we were able to add more variables relevant to childhood traumas in the present study, compared with our previous report [3]. Since these trauma variables were from two different data sources (i.e., the EFTF interview and the 2015 LHMS), there was a slight difference in age frame in the questions (18 years old vs. age 16); for consistency, we assumed that all these traumatic events occurred in childhood. Each variable for childhood trauma was coded as a dichotomous variable based on the answer (0=no; 1=yes). To get a summarized score in the cluster dendrogram (see details below), we simply summed them in an unweighted way as a total score (possible range 0–10). Higher scores represent more severer traumas.

Childhood health: In addition to self-reported health, a widely used indicator of a person’s overall health status, we included two other common and important events in childhood: disabled for six months and head injury, because they were treated as measures of early life physical health [4, 6].

***Adulthood SES:***

In addition to education and total wealth, two common indicators of SES, we included other variables to more fully capture adulthood SES, including ever received Medicaid, ever received food stamps, proportion experiencing unemployment, satisfaction with present financial situation, and difficulties meeting payments on bills. Some of these variables have been included in previous literature [7]. Total wealth was derived by the RAND HRS groups and was reported in nominal dollars. It represents the net value of total wealth less IRA and all debt. Due to the wide range of its value, we categorized participants into five groups based on quintiles of total wealth. Because persons may experience multiple unemployment and there might be cumulative effect, we defined a variable called proportion of experiencing unemployment (range 0-1) based on the response from each wave, representing the percentage of survey waves when the participant experienced unemployment, with higher values representing more unemployment. For the other variables, we recoded them according to the response to the corresponding questions, e.g., a dichotomous variable for ever received Medicaid and a 5-point scale for difficulties for meeting payments on bills.

***Adulthood adversity:***

We included six measures for adulthood adversity, including adulthood traumas, neighborhood physical disorder, life time discrimination, chronic stressors, and life events, and major events with satisfied validity [8]. All the variables for the five measures (except major events) were from the psychosocial questionnaire in the EFTF Interview (2006-2016). More specifically, the seven dichotomized variables for adulthood traumas came from a previous paper by Krause et al [9, 10] and was included in a prior paper in HRS [7]. For the neighborhood physical disorder, four variables with a scale ranging from 1 to 7 were included. We had six dichotomized variables for life time discrimination [11, 12]. We had eight variables with a scale ranging from 1 to 4 for chronic stressors [13, 14]. We had five dichotomized variables for life events [15] and major events (assembled from the 2015 LHMS), respectively.

In the cluster dendrogram, we calculated a summarized score for these five measures except major events) based on the document used in HRS EFTF Interview [8]. For neighborhood physical disorder, we averaged the score across four variables. For other four measures, we simply summed them in an unweighted way as a total score. The range of these summarized scores were 0 to 7 for adulthood traumas, 1 to 7 for neighborhood physical disorder, 0 to 6 for life time discrimination, 8 to 32 for chronic stressors, and 0 to 5 for stressful life events.

**Behaviors:**

As mentioned in the main text, in 1 behaviors domain, we included four major behavioral factors that are relevant to health, including obesity, smoking, alcohol consumption, and physical activity. The definition of obesity and smoking have been provided in the main text. For alcohol consumption, due to no agreement on the definition and avoid artificial assignment, we simply kept the three variables in RAND HRS files including ever drinking, drinking days per week, and numbers of drinks per day. Similarly, we simply kept the six variables (from the 2015 LHMS) for physical activity, including vigorous and moderate activities from 18 to 49 years. Note that although most of behavioral factors occur in adulthood, they were not essentially the focus of this study; thus, they were not included into the concept of “childhood and adulthood circumstances” but as an important separate domain.

**Genetic factors**

All phenotypes for the five domains for genetic factors are listed below, and more information can be found in the document from HRS [16]. We controlled for potential population stratification [17].

| **Polygenic score (PGS)** | **Traits/phenotypes** |
| --- | --- |
| Anthropometric | Height |
|  | Body mass index |
|  | Waist circumference |
|  | Waist-to-Hip ratio |
| Disease/longevity | Coronary artery disease |
|  | Myocardial infarction |
|  | Type II diabetes |
|  | Alzheimer's disease |
|  | Longevity |
| Mental health/personality | Attention deficit/hyperactivity disorder PGC 2010 |
|  | Attention deficit/hyperactivity disorder PGC 2017 |
|  | Autism |
|  | Bipolar disorder |
|  | Mental health cross disorder |
|  | Major depressive disorder |
|  | Subjective wellbeing |
|  | Neuroticism |
|  | Depressive symptoms |
|  | Schizophrenia |
|  | Extraversion |
| Education/cognition | Educational attainment |
|  | General cognition |
| Smoking | Smoking initiation (ever/never) |
|  | Number of cigarettes smoked per day |

**Statistical Analyses**

***The Shapley Value Decomposition approach***

The Shapley Value Decomposition (Shapley approach hereafter) applied in this study is regression based. Besides testing for statistical significance of specific independent variables, the Shapley approach enables researchers to measure the shares (and their confidence intervals) of variation in health outcomes attributable to respective life course factors or domains of such life course factors. This section introduces the application of Shapley approach to this study.

The main outcome of interest in this study is the phenotypic age acceleration (*PhenoAgeAccel*), which represents phenotypic aging after accounting for chronological age. As a measure of the extent of *PhenoAgeAccel* inequality, the mean logarithmic deviation (MLD) of a *PhenoAgeAccel* distribution $F(PhenoAgeAccel)$, i.e. $MLD\left( F \right)$, denotes the total inequality of outcomes. MLD is a popular measure of inequality in statistics and econometrics and has been used to measure multiple dimensions of well-being [18-22]. MLD, a special case of the Generalized Entropy, a well-accepted family of inequality measures, enables us to fully decompose variations in health outcomes into observed and unobserved factors [23-26]. As suggested by Roemer and Trannoy [27], application of the Shapley approach to other continuous measures, such as phenotypic aging in our study, is a natural extension.

$$MLD=\frac{1}{N}\sum_{i=1}^{N} \left( \ln\bar{x}-lnx_{i} \right)=ln\bar{x}-\bar{lnx}$$

where N is the number of individuals, $x_{i}$ is health status of individual i. $\bar{x}$ is the mean of $x_{i}$. $\bar{lnx}$ is the mean of $\ln x_{i}$. The logarithmic form has several good features, among them the most relevant to us include: 1) it considers aversion to higher health inequality. Given the average phenotypic aging $\bar{x}$ (i.e. the first term of MLD fixed), more unequal health status corresponds to a larger second term and therefore higher MLD (Let us suppose there are two individuals with logarithmic values of phenotypic aging $\ln x_{1}$ and $\ln x_{2}$ respectively. According to *Jensen's Inequality*, a mathematical theorem, the second term increases with inequality $-(lnx_{1}+lnx_{2})/2>-\ln[{(x}_{1}+x_{2})/2]$); 2) the logarithmic form enables us to mitigate the possible overinfluence of outlier values.

The MLD is defined as the mean deviation of $\ln x_{i}$ from $\ln\bar{x}$. In other words, it measures the average difference between $\ln\bar{x}$ and $\ln x_{i}$. The MLD is nonnegative, takes the value zero when everyone has the same health status, and takes larger positive values as health becomes more unequal. The reason of using MLD in the Shapley decomposition is that it nicely distinguishes variation in health outcomes due to life course factors from that due to unobserved factors [27] and therefore facilitates cross-study comparisons.

The regression-based Shapley approach essentially divides the sample into types of individuals, each type corresponding to the set of individuals with the same value of independent variables (i.e., life course factors in this study). Each type is characterized by its own *PhenoAgeAccel* distribution. Let the type distributions be $\left\{ F^{t}(PhenoAgeAccel), t \epsilon T \right\}$ where $T$ is the set of types, and let type $t$ have frequency $f_{t}$ in the population and mean *PhenoAgeAccel* $\mu^{t}$, summarized by the vectors $f=f^{1}, \ldots, f^{T}$ and $\mu=\mu^{1}, \ldots, \mu^{T}$. We can construct a hypothetical distribution, denoted by $\Phi_{(\mu, f)}$, in which all members of each type $t$ receive the mean *PhenoAgeAccel* $\mu^{t}$ of that type. $\Phi_{(\mu, f)}$ has a cumulative distribution function that is a step function, with as many steps as types; it is often called the ‘smoothed’ distribution of $F$ associated with the typology $(f,\mu)$. MLD of total inequality is decomposable as follows:

$MLD\left( F \right)= MLD\left( \Phi\right)+ \sum_{t=1}^{T} f_{t}MLD(F_{t})$ (1)

A MLD can be fully decomposed into inequality due to observable life course factors (the first term on the right-hand side) and other unobserved characteristics (the second term on the right-hand side). Therefore, the ratio r measures the extent to which phenotypic age inequality is due to observed life course factors

$$r=\frac{MLD(\Phi)}{MLD(F)}$$

**A Simple Illustrating Example**: Suppose we have two binary childhood and adulthood circumstances in total, i.e. parental education (high/low) and adulthood financial hardship (no/yes). Therefore, there are four types, i.e. (high, no), (high, yes), (low, no), (low, yes). All individuals are partitioned into these four groups. Let’s assume for now all individuals within each type have the same value of *PhenoAgeAccel*, meaning that people with the same childhood and adulthood circumstances have the same level of phenotypic age acceleration, then the variation across the four types in *PhenoAgeAccel* can only be due to differences in childhood and adulthood circumstances. This variation is the numerator. The overall variation in *PhenoAgeAccel* across all individuals is the denominator. The ratio of the two measures proportion of health inequality that can be explained by observable childhood and adulthood circumstances.

Note that while this illustrating example only considers two life course factors for simplicity, in the implementation of this study, we used regression-based Shapley approach to consider more than 90 life course factors at the same time.

Conditional on using a rich set of life course factors, our regression-based Shapley approach follows procedures from [Ferreira and Gignoux](#_bookmark72) [19, 20], Niehues and Peichl [28] and Roemer and Trannoy [27] to specify the model

$ln {PhenoAgeAccel}_{i} = \boldsymbol{\alpha}\boldsymbol{V}_{\boldsymbol{i}}+ u_{i}$, (2)

*i* represents individual i. Since the MLD decomposition only allows positive health outcome measures (due to its component of logarithmic form) but is scale invariant, therefore we transform all values of *PhenoAgeAccel* to be positive by adding a positive number without altering its distribution. We tested multiple such positive values to make sure the results are robust to our transformation and remain essentially the same.

This reduced form estimation enables us to derive the overall fraction of variance in *PhenoAgeAccel* that is explained by a vector $\boldsymbol{V}_{\boldsymbol{i}}$ of childhood ($\boldsymbol{C}_{\boldsymbol{i}}$) and adulthood ($\boldsymbol{A}_{\boldsymbol{i}}$) factors, where $\boldsymbol{V=}\left( \boldsymbol{C,A} \right)$. Based on this estimation, we construct a parametric estimate of the smoothed distribution $\Phi$ defined earlier by replacing *PhenoAgeAccel* outcomes by their predictions:

$\hat{PhenoAgeAccel}=exp(\hat{\boldsymbol{\alpha}}\boldsymbol{V}_{\boldsymbol{i}}\boldsymbol{)}$ (3)

Let $\Phi$ be the distribution of estimated *PhenoAgeAccel*. In this counterfactual, all individuals with the same childhood and adulthood characteristics have the same *PhenoAgeAccel*. Thus $r$ can be rewritten as:

$$r=\frac{MLD(\left\{ \hat{PhenoAgeAccel} \right\})}{MLD(\left\{ PhenoAgeAccel \right\})}$$

The overall contribution $\hat{r}$ can be neatly decomposed into components for each category $\hat{r}^{j}$in the childhood and adulthood vector ***V***.

$\hat{r}=\sum_{j} \hat{r}^{j}=\sum_{j} {(var PhenoAgeAccel)}^{-1}[a_{j}^{2}var V^{j}+\frac{1}{2}\sum_{k} a_{k}a_{j}cov\left( V^{k},V^{j} \right)]$ (4)

where *j*, *k*=1, 2, … as categories of childhood and adulthood characteristics. $\alpha_{j}$and $\alpha_{k}$ are coefficients of categories *j* and *k* in equation (2). Equation (4) presents an example of a Shapley Value Decomposition. This approach provides an appropriate way to assign roles to sources in generating health inequality [20, 22-24, 27].

A particular category *j*’s overall contribution to the variance in *PhenoAgeAccel* – $\hat{r}^{j}$ – corresponds to an average between two channels. Intuitively, childhood circumstances may not only directly impact health in old age, but exert their effects indirectly through shaping other childhood and adulthood characteristics. Formally, all $V_{j\neq J}^{j}$ are held constant in the *direct* contribution of category *j*, i.e. $a_{j}^{2}{(var PhenoAgeAccel)}^{-1}var V^{j}$. Regarding the *indirect* contribution, category *j* itself is held constant, and its indirect contribution, i.e. $\frac{1}{2}{(var PhenoAgeAccel)}^{-1}\sum_{k} a_{k}a_{j}cov\left( V^{k},V^{j} \right)$, is taken as the difference between the total variance and the ensuing variance.

To compute the Shapley Value Decomposition, we first estimate the inequality measure for all possible permutations of the childhood and adulthood variables (domains in the present study). In a second step, the average marginal effect of each domain on inequality in *PhenoAgeAccel* is computed [25]. This procedure is very computationally intensive as 2^K^ (K= number of domains) must be computed. Finally, we repeat these steps to obtain the bootstrap standard errors.

There are substantial advantages compared to other decomposition methods. First, it is order independent, meaning that the order of characteristics for decomposition does not affect the results. Second, it is additive, meaning that the sum of each domain’s contribution to inequality in *PhenoAgeAccel* adds up to the total value of overall contribution to inequality in *PhenoAgeAccel*. Though the decomposition should not be seen as causal, it offers an idea of the relative importance of factors [20]. Third, the Shapley approach has been a basic built-in toolkit in popular statistical packages, such as STATA and R, which further simplifies the analysis and dissemination.

It is noteworthy that this approach may provide a lower-bound estimate of contribution to health inequality, because variations in health may not be all accounted for due to *unobserved* characteristics. To obtain population-based estimates, we incorporated survey weights in VBS 2016 in this analysis. Moreover, the function form of MLD implicitly assumes increasing disutility from more phenotypic age acceleration. Our results are robust to variance decomposition, an alternative decomposition method that assumes no specific curvature of functional form. See [25] for an illustration of variance decomposition and its comparisons with MLD decomposition.

***Principal components analysis (PCA) and hierarchal cluster analysis (HCA)***

To reduce the dimensionality of potential correlated variables for childhood and adulthood circumstances, we performed principal components analysis (PCA), a statistical procedure that converts possibly correlated variables into a set of values of linearly uncorrelated variables named PCs. A total of 60 (mainly categorical) variables from domains of childhood SES, childhood adversity, adulthood SES, and adulthood adversity were included and finally the top four PCs were selected via scree plot (seeking for a “break” between the principal components with relatively large eigenvalues and those with small eigenvalues [29]), explaining 22.4% of total variance.

To identify subpopulations that phenotypically age at different level, we performed a hierarchal clustering analysis (HCA) for all participants using the top four PCs from PCA with the WGCNA R package [30]. As a result, we obtained six optimal subpopulations/clusters using a cut height of 0.70 and a minimum size of 20. These subpopulations/clusters were represented by different colors: "blue", "green", "turquoise", "orange, "yellow", and “red”. To show which characteristic these subpopulations/clusters represented or how they differed in characteristics, we then categorized several main circumstances including race/ethnicity, parental education, self-reported family poverty, childhood traumas (summarized score), adulthood traumas (summarized score), neighborhood physical disorder (summarized score), life time discrimination (summarized score), chronic stressors (summarized score), life events (summarized score), education, and total wealth, and draw the cluster dendrogram accompanied by these categories. To keep consistent, all values of these categories were indicated by different colors. In this cluster dendrogram, the closer to “darkorange”, the higher dose of exposing to risk factors (except race/ethnicity). For example, parental education more than 16 years was indicated by “darkmagenta”, whereas parental education less than 12 years was indicated by “darkorange”. Similar to prior work using WGCNA [30], we calculated a quantitative continuous measure as the membership of these clusters as mentioned in the main text. We examined the correlation between these subpopulations/clusters using these cluster membership values. Other analyses including comparisons of the PhenoAgeAccel among subpopulations/clusters and examining associations of these subpopulations/clusters (using the cluster membership values), behaviors, and genetics with PhenoAge are described in the main text.

To test the robustness of our results, we completed three sets of sensitivity analyses: 1) to test the results were robust with respect to fasting, we reran all our analyses in those who fasted (n=1,579 for the Shapley Value Decomposition analysis); 2) since most of the adulthood adversity variables had missing data around 130 while other variables had relative small numbers of missingness, we simply imputed the missingness for adulthood adversity variables with median value and rerun our analyses with corresponding dummy variables [31]; 3) because no survey weight was available in the 2015 LHMS, we used alternative weight from the main survey. All analyses were performed using SAS version 9.4 (SAS Institute, Cary, NC), R and STATA version 14.0 software (Stata Corporation, College Station, TX). P-values < 0.05 were considered as statistically significant.

# **Results**

**Sensitivity analyses**

# Sensitivity analyses revealed comparable results for the contribution of life circumstances to PhenoAgeAccel when 1) limiting the sample to those who fasted at least 8 hours (S5 Fig and S6 Fig); 2) when using dummy variables and imputed values for missing data on adulthood adversity; and 3) when using alternative weights from the main survey.

**References**

1. Sonnega A, Faul JD, Ofstedal MB, Langa KM, Phillips JW, Weir DR. Cohort Profile: the Health and Retirement Study (HRS). Int J Epidemiol. 2014;43(2):576-85. doi: 10.1093/ije/dyu067. PMID: 24671021.

2. Crimmins E, Faul J, Thyagarajan B, Weir D. Venous blood collection and assay protocol in the 2016 Health and Retirement Study: 2016 Venous Blood Study (VBS). Ann Arbor: MI: Institute for Social Research, University of Michigan, 2017.

3. Levine ME, Cole SW, Weir DR, Crimmins EM. Childhood and later life stressors and increased inflammatory gene expression at older ages. Soc Sci Med. 2015;130:16-22. doi: 10.1016/j.socscimed.2015.01.030. PMID: 25658624.

4. Latham K. The "long arm" of childhood health: linking childhood disability to late midlife mental health. Res Aging. 2015;37(1):82-102. doi: 10.1177/0164027514522276. PMID: 25651552.

5. Papageorge N, Thom K. Genes, education, and labor market outcomes: evidence from the health and retirement study. Upjohn Institute Working Paper. 2017:17-232. doi: 10.17848/wp17-273.

6. Bramlett HM, Dietrich WD. Long-Term Consequences of Traumatic Brain Injury: Current Status of Potential Mechanisms of Injury and Neurological Outcomes. J Neurotrauma. 2015;32(23):1834-48. doi: 10.1089/neu.2014.3352. PMID: 25158206.

7. Puterman E, Gemmill A, Karasek D, Weir D, Adler NE, Prather AA, et al. Lifespan adversity and later adulthood telomere length in the nationally representative US Health and Retirement Study. Proc Natl Acad Sci U S A. 2016;113(42):E6335-E42. doi: 10.1073/pnas.1525602113. PMID: 27698131.

8. Clarke P, Fisher G, House J, Smith J, Weir D. Guide to content of the HRS psychosocial leave-behind participant lifestyle questionnaires: 2004 & 2006. Survey Research Center, Institute for Social Research. 2008.

9. Krause N, Shaw BA, Cairney J. A descriptive epidemiology of lifetime trauma and the physical health status of older adults. Psychol Aging. 2004;19(4):637-48. doi: 10.1037/0882-7974.19.4.637. PMID: 15584789.

10. Turner RJ, Lloyd DA. Lifetime traumas and mental health: the significance of cumulative adversity. J Health Soc Behav. 1995;36(4):360-76. PMID: 8719054.

11. Williams DR, Yan Y, Jackson JS, Anderson NB. Racial Differences in Physical and Mental Health: Socio-economic Status, Stress and Discrimination. J Health Psychol. 1997;2(3):335-51. doi: 10.1177/135910539700200305. PMID: 22013026.

12. Kessler RC, Mickelson KD, Williams DR. The prevalence, distribution, and mental health correlates of perceived discrimination in the United States. J Health Soc Behav. 1999;40(3):208-30. PMID: 10513145.

13. Troxel WM, Matthews KA, Bromberger JT, Sutton-Tyrrell K. Chronic stress burden, discrimination, and subclinical carotid artery disease in African American and Caucasian women. Health Psychol. 2003;22(3):300-9. PMID: 12790258.

14. Bromberger JT, Matthews KA. A longitudinal study of the effects of pessimism, trait anxiety, and life stress on depressive symptoms in middle-aged women. Psychol Aging. 1996;11(2):207-13. PMID: 8795049.

15. Turner RJ, Wheaton B, Lloyd DA. The Epidemiology of Social Stress. Am Sociol Rev. 1995;60(1):104-25. doi: 10.2307/2096348.

16. Erin BW, Lauren LS, Jessica DF. HRS Polygenic Scores: 2006-2010 Genetic Data. Ann Arbor, Michigan: Survey Research Center, Institute for Social Research, University of Michigan, 2017.

17. Price AL, Patterson NJ, Plenge RM, Weinblatt ME, Shadick NA, Reich D. Principal components analysis corrects for stratification in genome-wide association studies. Nat Genet. 2006;38(8):904-9. doi: 10.1038/ng1847. PMID: 16862161.

18. Hufe P, Peichl A, Roemer J, Ungerer M. Inequality of income acquisition: the role of childhood circumstances. Soc Choice Welf. 2017;49(3):499-544. doi: 10.1007/s00355-017-1044-x.

19. Ferreira F, Gignoux J. The measurement of inequality of opportunity: theory and an application to Latin America. Rev Income Wealth. 2011;57(4):622-57. doi: 10.1111/j.1475-4991.2011.00467.x.

20. Ferreira F, Gignoux J. The Measurement of Educational Inequality: Achievement and Opportunity. World Bank Econ Rev. 2013;28:210-46. doi: 10.1596/1813-9450-5873.

21. Haughton J, Khandker SR. Handbook on poverty and inequality. Washington, DC: World Bank; 2009.

22. Björklund A, Jäntti M, Roemer JE. Equality of opportunity and the distribution of long-run income in Sweden. Soc Choice Welf. 2012;39(2):675-96. doi: 10.1007/s00355-011-0609-3.

23. Shorrocks AF. Decomposition procedures for distributional analysis: a unified framework based on the Shapley value. J Econ Inequal. 2013;11(1):99-126. doi: 10.1007/s10888-011-9214-z.

24. Jusot F, Tubeuf S, Trannoy A. Circumstances and efforts: how important is their correlation for the measurement of inequality of opportunity in health? Health Econ. 2013;22(12):1470-95. doi: 10.1002/hec.2896. PMID: 23345011.

25. Juárez FWC, Soloaga I. iop: Estimating ex-ante inequality of opportunity. Stata J. 2014;14(4):830-46. doi: 10.1177/1536867X1401400408.

26. Shorrocks AF. The class of additively decomposable inequality measures. Econometrica. 1980:613-25. doi: 10.2307/1913126.

27. Roemer J, Trannoy A. Equality of Opportunity: Theory and Measurement. J Econ Lit. 2016;54(4):1288-332. doi: 10.1002/hec.3092. PMID: 25073459.

28. Niehues J, Peichl A. Upper bounds of inequality of opportunity: theory and evidence for Germany and the US. Soc Choice Welf. 2014;43(1):73-99. doi: 10.1007/s00355-013-0770-y.

29. Cattell RB. The Scree Test For The Number Of Factors. Multivariate Behav Res. 1966;1(2):245-76. doi: 10.1207/s15327906mbr0102_10. PMID: 26828106.

30. Langfelder P, Horvath S. WGCNA: an R package for weighted correlation network analysis. BMC Bioinformatics. 2008;9:559. doi: 10.1186/1471-2105-9-559. PMID: 19114008.

31. Allison PD. Missing data. Thousand Oaks, Calif: Sage publications; 2001.
