# Supplementary figures and images for: Associations of genetics, behaviors, and life course circumstances with a novel aging and healthspan measure: Evidence from the Health and Retirement Study
Source: PLoS Med. 2019 Jun 18;16(6):e1002827. doi: 10.1371/journal.pmed.1002827 (PMC6581243; doi:10.1371/journal.pmed.1002827)

### Cluster Dendrogram

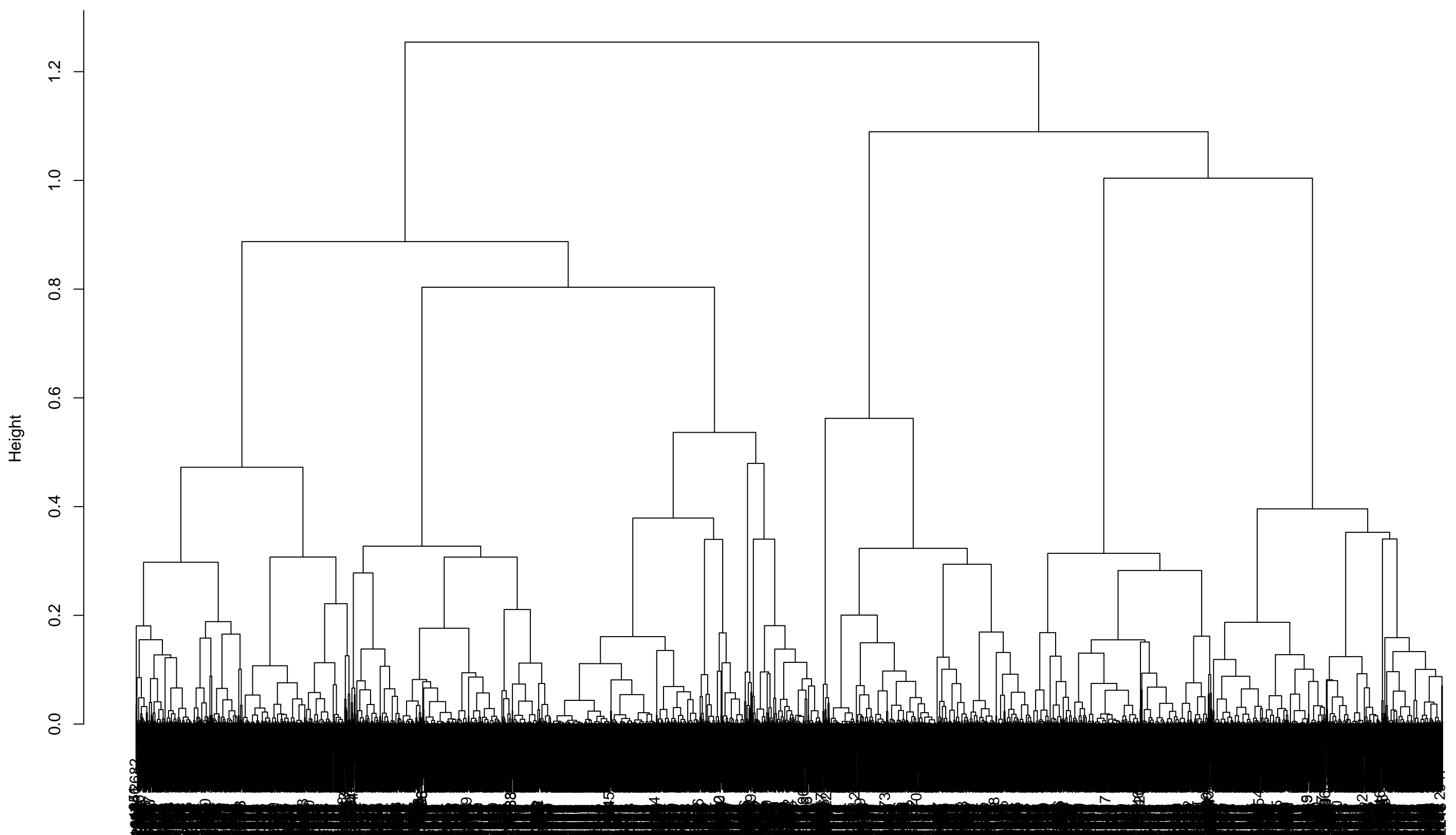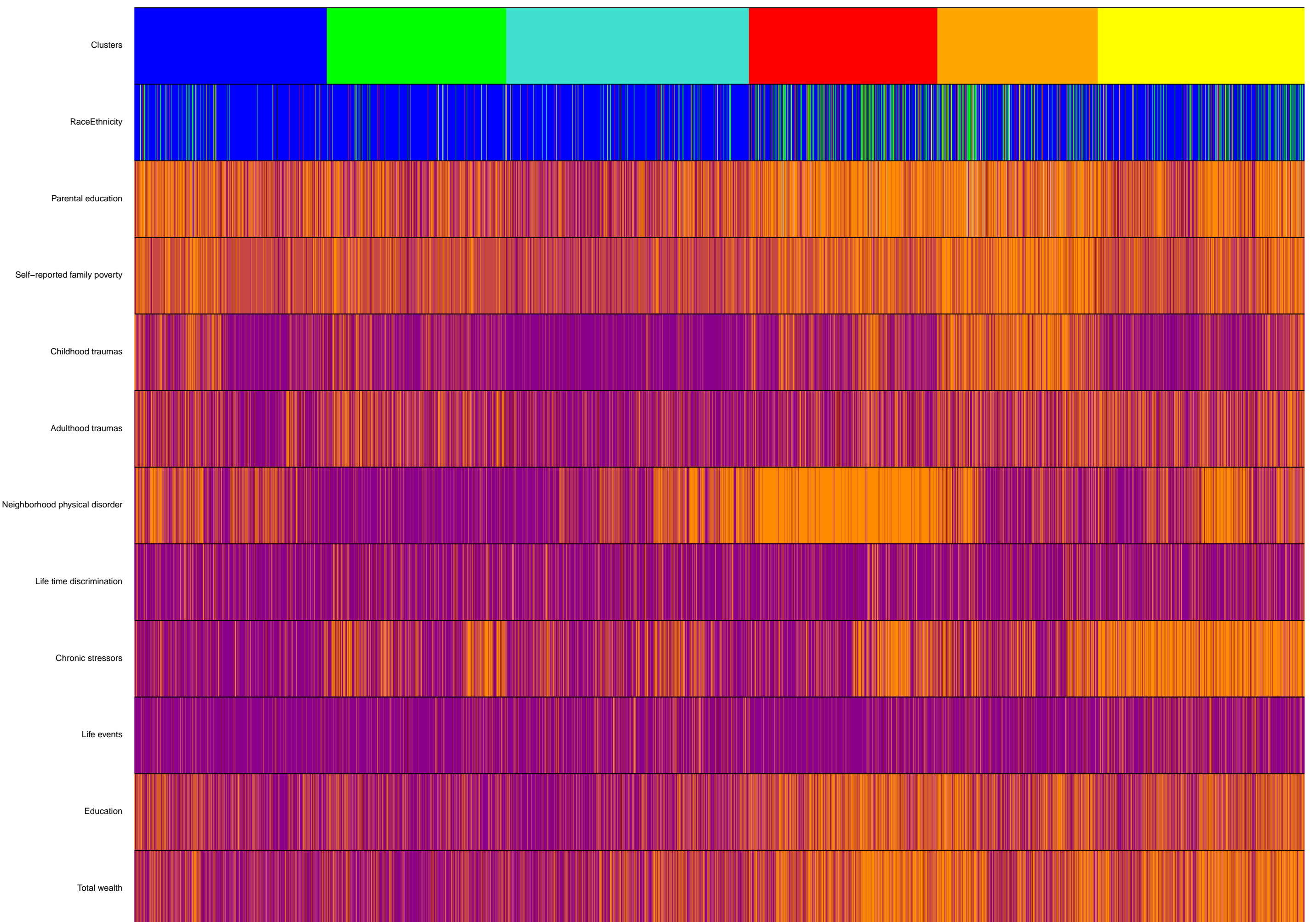

Supplement: S1 Fig — Six subpopulations/clusters were identified, labeled “blue” (n = 470), “green” (n = 438), “turquoise” (n = 593), “orange” (n = 392), “yellow” (n = 503), and “red” (n = 460). For the categories of summarized measures given in the lower half of the figure, the closer to “dark orange,” the higher the dose of exposing to this risk factor (except race/ethnicity). For example, parental education more than 16 years was indicated by “dark magenta,” whereas parental education less than 12 years was indicated by “dark orange.” (PDF) [file pmed.1002827.s003.pdf]

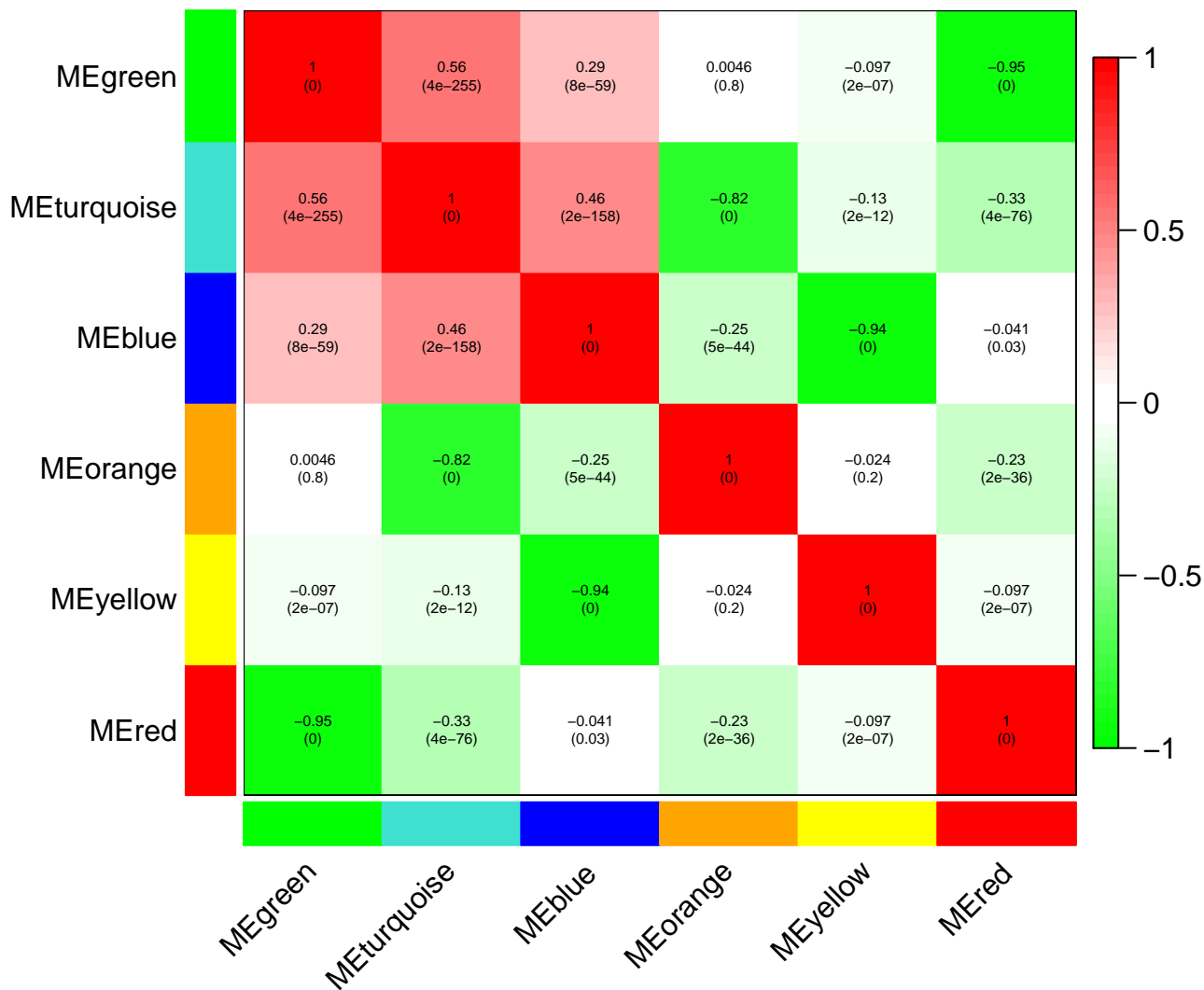

Supplement: S2 Fig — To determine what each group represents, we calculated a continuous measure (cluster membership) for each cluster (between −1 and 1) that denotes how strongly a person belongs to that given cluster—for instance, someone may have a score of 0.8 for the green cluster and −0.6 for the red cluster, suggesting he/she is very similar to the profile represented by the green cluster, but not that by the red cluster. Each cell reports the correlation (and p-value) between cluster memberships. (PDF) [file pmed.1002827.s004.pdf]

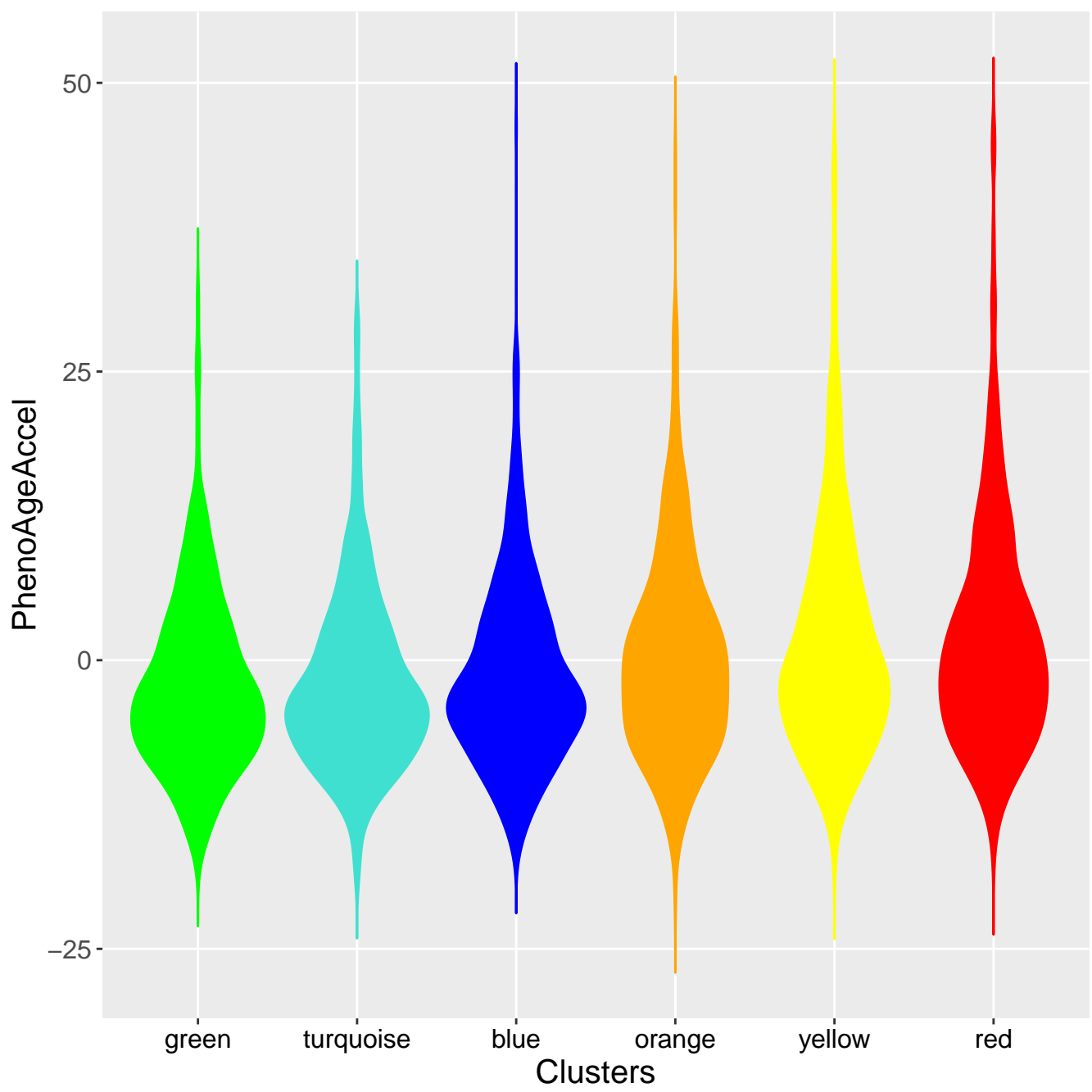

Supplement: S3 Fig — (PDF) [file pmed.1002827.s005.pdf]

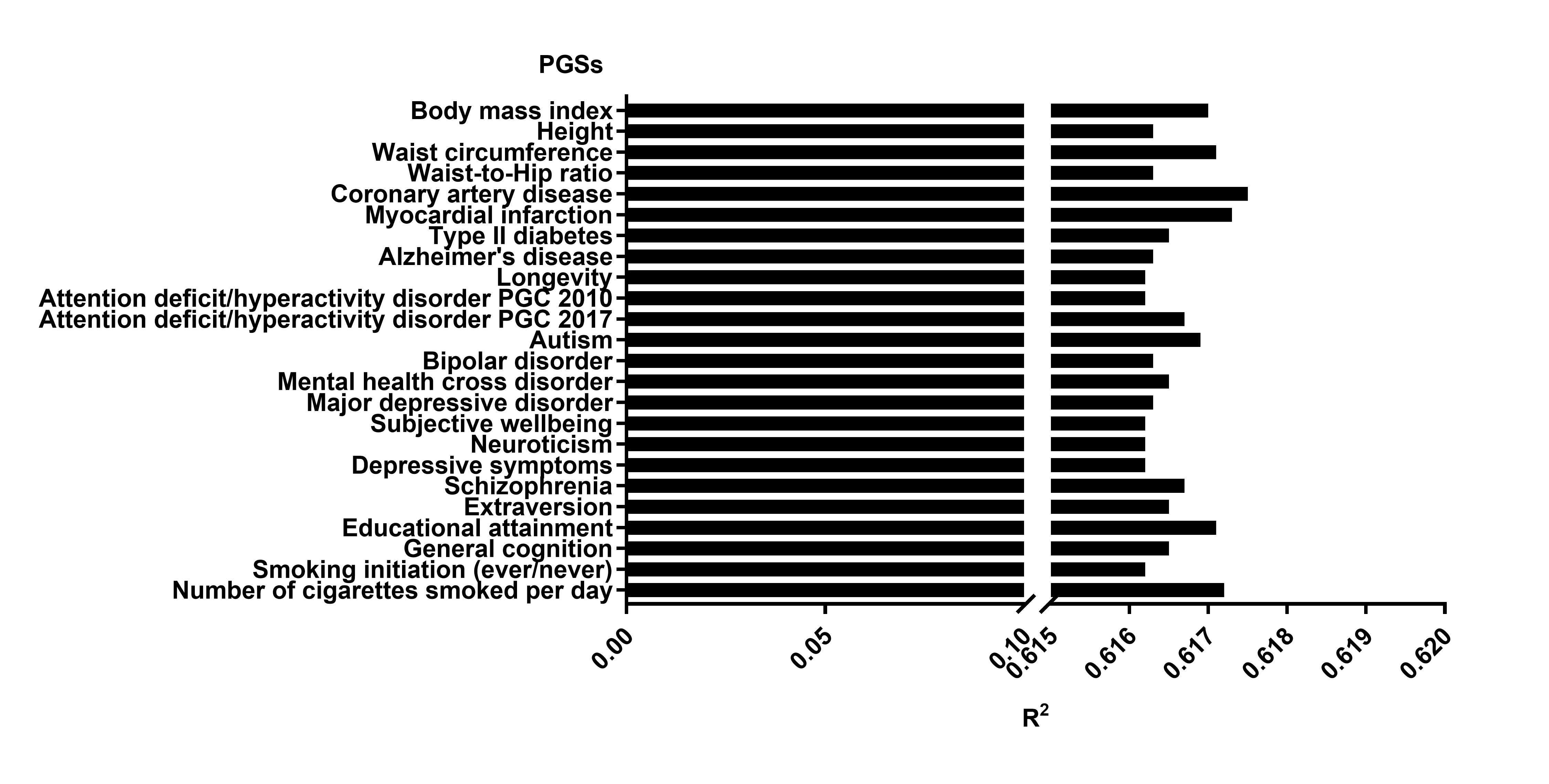

Supplement: S4 Fig — (TIF) [file pmed.1002827.s006.tif]

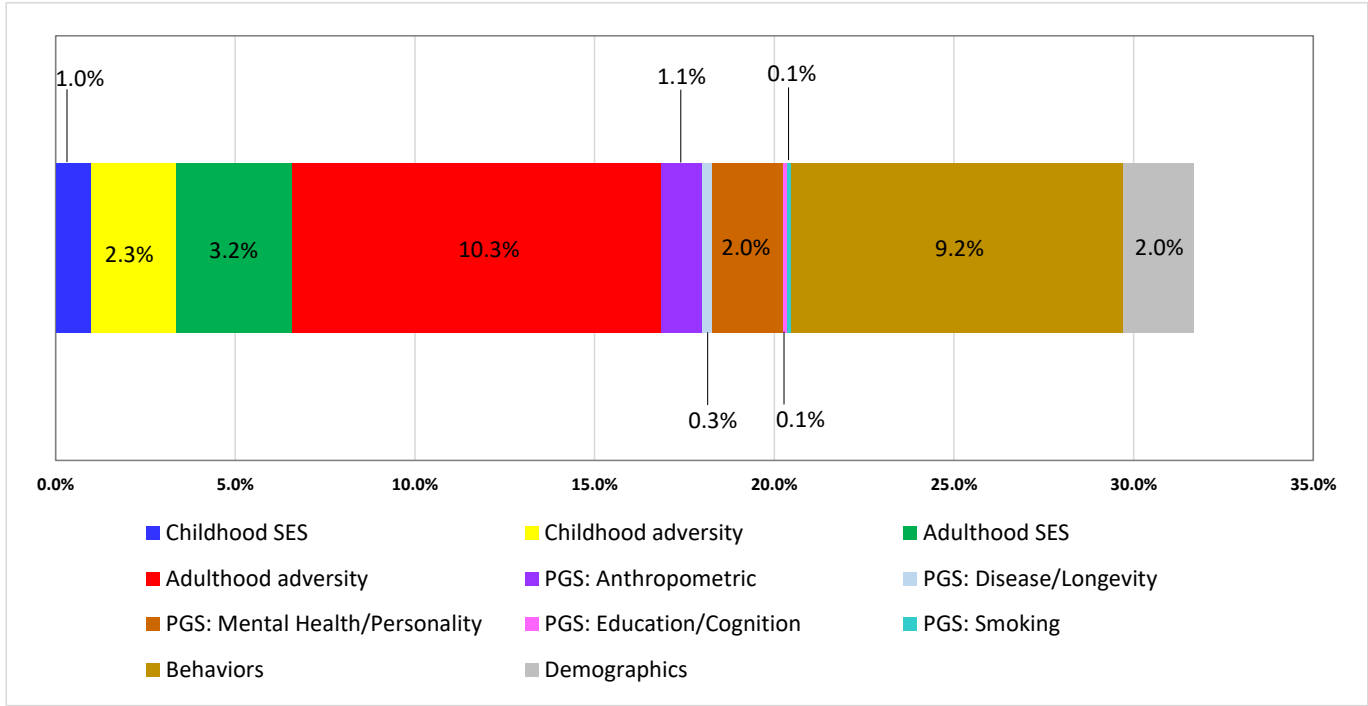

Supplement: S5 Fig — Overall, all the 11 domains contributed 31.7% (bootstrap standard error = 0.004) of variance in PhenoAgeAccel. (PDF) [file pmed.1002827.s007.pdf]

**p = 2.2e-11**

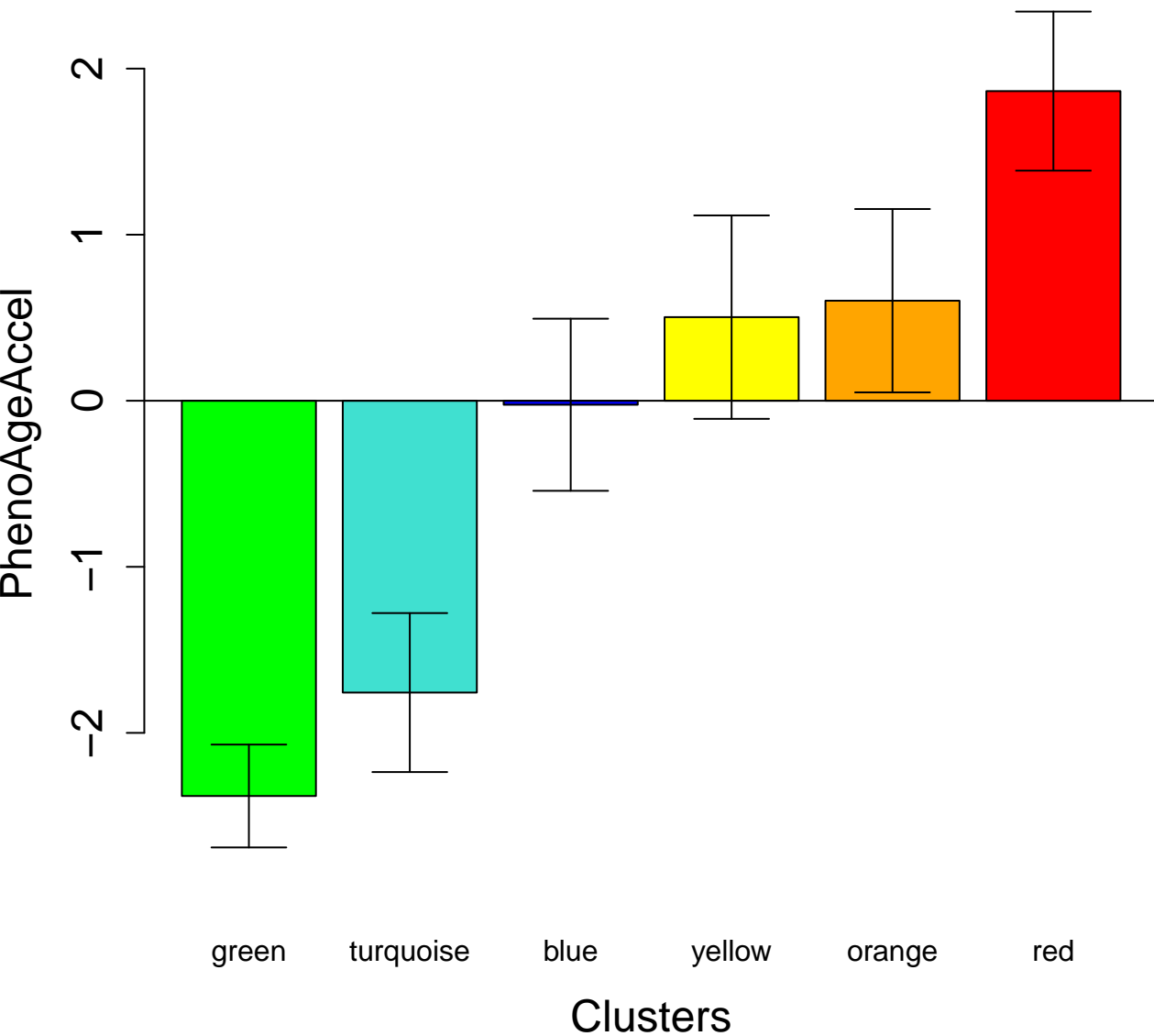

Supplement: S6 Fig — (PDF) [file pmed.1002827.s008.pdf]
