# Supplementary material for: Associations of genetics, behaviors, and life course circumstances with a novel aging and healthspan measure: Evidence from the Health and Retirement Study
Source: PLoS Med. 2019 Jun 18;16(6):e1002827. doi: 10.1371/journal.pmed.1002827 (PMC6581243; doi:10.1371/journal.pmed.1002827)
Supplement: S1 Table — (DOCX) [file pmed.1002827.s009.docx]

**S1 Table. Questions and responses for variables included in the childhood and adulthood circumstances and behaviors**

|  | | **Variables names** | **Questions** | **Responses/description** |
| --- | --- | --- | --- | --- |
| **Childhood circumstances** | | |  |  |
|  | **Childhood SES** | |  |  |
|  |  | Relocated due to financial difficulties | While you were growing up, before age 16, did financial difficulties ever cause you or your family to move to a different place? | 0=no;1=yes |
|  |  | Family received financial help | Before age 16, was there a time when you or your family received help from relatives because of financial difficulties? | 0=no;1=yes |
|  |  | Self-reported family poverty | Would you say your family during that time (i.e. from birth to age 16) was pretty well off financially, about average, or poor? | 0= pretty well off financially; 1=about average; 2=poor |
|  |  | Parental education | the highest grade of school your father or mother completed | Cont. |
|  |  | Father lose jobs | Before age 16, was there a time of several months or more when your father had no job? | 0=no;1=yes; 2=father never worked/always disabled; 3= never lived with father/father was not alive |
|  | **Childhood adversity** | |  |  |
|  | **Childhood traumas** | |  |  |
|  |  | Trouble with police | Before you were 18 years old, were you ever in trouble with the police? | 0=no;1=yes |
|  |  | Repeated school | Before you were 18 years old, did you have to do a year of school over again? | 0=no;1=yes |
|  |  | Physically abused | Before you were 18 years old, were you ever physically abused by either of your parents? | 0=no;1=yes |
|  |  | Parents used drugs or alcohol | Before you were 18 years old, did either of your parents drink or use drugs so often that it caused problems in the family? | 0=no;1=yes |
|  |  | Parents died | Before age 16 did you one or both parents die? | 0=no;1=yes |
|  |  | Separated from mother | Before age 16 did you ever separated from you mother for 6 months or longer? | 0=no;1=yes |
|  |  | Separated from father | Before age 16 did you ever separated from you father for 6 months or longer? | 0=no;1=yes |
|  |  | Live in orphanage | Before you were age 16 did you ever live in a children's home or orphanage? | 0=no;1=yes |
|  |  | Live in a foster home | Before you were age 16 did you ever live with a foster family or in a foster home? | 0=no;1=yes |
|  |  | Parents separated or divorced | Before you were age 16 did your biological or adoptive parents separate or divorce? | 0=no;1=yes |
|  | Childhood health | |  |  |
|  |  | Self-reported health | Would you say that your health during that time was excellent, very good, good, fair, or poor? | 1.=excellent; 2=very good; 3=good; 4=fair; 5=poor |
|  |  | Disabled for six months, yes | Before you were 16 years old, were you ever disabled for six months or more because of a health problem? That is, were you unable to do the usual activities of classmates or other children your age? | 0=no;1=yes |
|  |  | Head injury, yes | Before you were 16 years old, did you have a blow to the head, a head injury or head trauma that was severe enough to require medical attention, to cause loss of consciousness or memory loss for a period of time? | 0=no;1=yes |
| **Adulthood circumstance** | | |  |  |
|  | **Adulthood SES** | |  |  |
|  |  | Education, years | The highest grade of school you completed | Cont. |
|  |  | Ever received Medicaid | Have you been covered by Medicaid health insurance | 0=no;1=yes |
|  |  | Ever received food stamps | Ever received food stamps | 0=no;1=yes |
|  |  | Proportion of experiencing unemployment | unemployed looking for work or temporarily laid off | Cont. (proportion) |
|  |  | Total wealth | Total Wealth (Excluding IRAs) | Five categories based on quintiles |
|  |  | Satisfaction with present financial situation | How satisfied are you with (your/your family's) present financial situation? | 1=completely satisfied; 2=very satisfied; 3=somewhat satisfied; 4=not very satisfied; 5=not at all satisfied |
|  |  | Difficulties for meeting payments on bills | How difficult is it for (you/your family) to meet monthly payments on (your/your family's) bills? | 1=not at all difficult; 2=not very difficult; 3=somewhat difficult; 4=very difficult; 5=completely difficult |
|  | **Adulthood adversity** | |  |  |
|  |  | **Adulthood traumas** | | |
|  |  | Experienced the death of a child | Has a child of yours ever died? | 0=no;1=yes |
|  |  | Experienced a natural disaster | Have you ever been in a major fire, flood, earthquake, or other natural disaster? | 0=no;1=yes |
|  |  | Fired a weapon in combat | Have you ever fired a weapon in combat or been fired upon in combat? | 0=no;1=yes |
|  |  | Been a victim of a physical attack | Were you the victim of a serious physical attack or assault in your life? | 0=no;1=yes |
|  |  | Ever had life-threatening illness | Did you ever have a life-threatening illness or accident? | 0=no;1=yes |
|  |  | Ever had a spouse or child with life-threatening illness | Did your spouse or a child of yours ever have a life-threatening illness or accident? | 0=no;1=yes |
|  |  | Spouse, partner, or child ever been addicted to drugs or alcohol? | Has your spouse, partner, or child ever been addicted to drugs or alcohol? | 0=no;1=yes |
|  |  | **Neighborhood physical disorder** | | |
|  |  | Had vandalism and graffiti | There is no problem with vandalism and graffiti in this area/Vandalism and graffiti are a big problem in this area | Cont. |
|  |  | Feel afraid to walk alone after dark | People feel safe walking alone in this area after dark/People would be afraid to walk alone in this area after dark | Cont. |
|  |  | Full of rubbish and litter | This area is kept very clean/This area is always full of rubbish and litter | Cont. |
|  |  | Had vacant or deserted houses | There are no vacant or deserted houses or storefronts in this area/There are many vacant or deserted houses or storefronts in this area | Cont. |
|  |  | **Life time discrimination** | | |
|  |  | Unfairly dismissed from a job | At any time in your life, have you ever been unfairly dismissed from a job? | 0=no;1=yes |
|  |  | Unfairly ever not been hired for a job | For unfair reasons, have you ever not been hired for a job? | 0=no;1=yes |
|  |  | Ever been unfairly denied a promotion | Have you ever been unfairly denied a promotion? | 0=no;1=yes |
|  |  | Unfairly been prevented from moving into a neighborhood | Have you ever been unfairly prevented from moving into a neighborhood because the landlord or a realtor refused to sell or rent you a house or apartment? | 0=no;1=yes |
|  |  | Ever been unfairly denied a bank loan | Have you ever been unfairly denied a bank loan? | 0=no;1=yes |
|  |  | Ever been unfairly treated by the police | Have you ever been unfairly stopped, searched, questioned, physically threatened or abused by the police? | 0=no;1=yes |
|  |  | **Chronic stressors** | | |
|  |  | Ongoing health problems (in yourself) | Ongoing health problems (in yourself) | 1=No, didn’t happen, 2=Yes, but not upsetting, 3=Yes, somewhat upsetting, 4=Yes, very upsetting |
|  |  | Ongoing physical or emotional problems (in spouse or child) | Ongoing physical or emotional problems (in spouse or child) | 1=No, didn’t happen, 2=Yes, but not upsetting, 3=Yes, somewhat upsetting, 4=Yes, very upsetting |
|  |  | Ongoing problems with alcohol or drug use in family member | Ongoing problems with alcohol or drug use in family member | 1=No, didn’t happen, 2=Yes, but not upsetting, 3=Yes, somewhat upsetting, 4=Yes, very upsetting |
|  |  | Ongoing difficulties at work | Ongoing difficulties at work | 1=No, didn’t happen, 2=Yes, but not upsetting, 3=Yes, somewhat upsetting, 4=Yes, very upsetting |
|  |  | Ongoing financial strain | Ongoing financial strain | 1=No, didn’t happen, 2=Yes, but not upsetting, 3=Yes, somewhat upsetting, 4=Yes, very upsetting |
|  |  | Ongoing housing problems | Ongoing housing problems | 1=No, didn’t happen, 2=Yes, but not upsetting, 3=Yes, somewhat upsetting, 4=Yes, very upsetting |
|  |  | Ongoing problems in a close relationship | Ongoing problems in a close relationship | 1=No, didn’t happen, 2=Yes, but not upsetting, 3=Yes, somewhat upsetting, 4=Yes, very upsetting |
|  |  | Helping at least one sick, limited, or frail family member or friend on a regular basis | Helping at least one sick, limited, or frail family member or friend on a regular basis. | 1=No, didn’t happen, 2=Yes, but not upsetting, 3=Yes, somewhat upsetting, 4=Yes, very upsetting |
|  |  | **Life events** | |  |
|  |  | Involuntarily lost a job for reasons other than retirement | Have you involuntarily lost a job for reasons other than retirement at any point in the past five years? | 0=no;1=yes |
|  |  | Unemployed | Have you been unemployed and looking for work for longer than 3 months at some point in the past five years? | 0=no;1=yes |
|  |  | Anyone else in your household unemployed | Was anyone else in your household unemployed and looking for work for longer than 3 months in the past five years? | 0=no;1=yes |
|  |  | Moved to a worse residence or neighborhood | Have you moved to a worse residence or neighborhood in the past five years? | 0=no;1=yes |
|  |  | Robbed or burglarized | Were you robbed or did you have your home burglarized in the past five years? | 0=no;1=yes |
|  |  | **Major events** | |  |
|  |  | Ever been in a jail | Before age 50, have you ever been in a jail, prison, or a detention center for more than 3 days? | 0=no;1=yes |
|  |  | Ever been in a hospital | Before age 50, have you ever been a long-term inpatient in a hospital for 1 month or more? | 0=no;1=yes |
|  |  | Ever lived in a combat zone | Before age 50, have you ever lived in a combat zone? | 0=no;1=yes |
|  |  | Ever lived in military housing | Before age 50, have you ever lived on a military base or in military housing? | 0=no;1=yes |
|  |  | Ever been homeless | Before age 50, have you ever been homeless for 1 month or more? | 0=no;1=yes |
|  | **Behaviors** | |  |  |
|  |  | Proportion obesity |  | Cont. (proportion) |
|  |  | Smoking |  | 0=Never smoking; 1=former smoking; 2=current smoking |
|  |  | Alcohol consumption |  |  |
|  |  | Ever drinking |  | 0=no;1=yes |
|  |  | Drinking days per week |  | Cont. |
|  |  | Numbers of drinks per drinking day |  | Cont. |
|  |  | Activities |  |  |
|  |  | Vigorous activities age 18–29 years | Between ages 18 and 29, how often did you take part in or train for sports or activities that are vigorous? | 0=no; 1=more than once a week* |
|  |  | Vigorous activities age 30–39 years | Between ages 30 and 39, how often did you take part in or train for sports or activities that are vigorous? | 0=no; 1=more than once a week* |
|  |  | Vigorous activities age 40–49 years | Between ages 40 and 49, how often did you take part in or train for sports or activities that are vigorous? | 0=no; 1=more than once a week* |
|  |  | moderate activities age 18–29 years | Between ages 18 and 29, how often did you take part in or train for sports or activities that are moderately energetic? | 0=no; 1=more than once a week* |
|  |  | moderate activities age 30–39 years | Between ages 30 and 39, how often did you take part in or train for sports or activities that are moderately energetic? | 0=no; 1=more than once a week* |
|  |  | moderate activities age 40–49 years | Between ages 40 and 49, how often did you take part in or train for sports or activities that are moderately energetic? | 0=no; 1=more than once a week* |

SES, socioeconomic status.

*To be consistent with other variables, the responses for activities were recoded based on raw responses (every day, more than once a week, once a week, one to three times a month, and hardly ever or never). We combined the first two as one category and others as another.
